# High-Affinity Anion Binding by Steroidal Squaramide Receptors**

**DOI:** 10.1002/anie.201411805

**Published:** 2015-02-17

**Authors:** Sophie J Edwards, Hennie Valkenier, Nathalie Busschaert, Philip A Gale, Anthony P Davis

**Affiliations:** School of Chemistry, University of Bristol, Cantock*s CloseBristol BS8 1TS (UK); Chemistry, University of SouthamptonSouthampton, SO17 1BJ (UK)

**Keywords:** anions, membranes, molecular recognition, ionophores, supramolecular chemistry

## Abstract

Exceptionally powerful anion receptors have been constructed by placing squaramide groups in axial positions on a steroidal framework. The steroid preorganizes the squaramide NH groups such that they can act cooperatively on a bound anion, while maintaining solubility in nonpolar media. The acidic NH groups confer higher affinities than previously-used ureas or thioureas. Binding constants exceeding 10^14^ m^−1^ have been measured for tetraethylammonium salts in chloroform by employing a variation of Cram’s extraction procedure. The receptors have also been studied as transmembrane anion carriers in unilamellar vesicles. Unusually their activities do not correlate with anion affinities, thus suggesting an upper limit for binding strength in the design of anion carriers.

Anion recognition has become a major theme of supramolecular chemistry.[1] An important motivation is the central role which anions play in biology. Most biomolecules have anionic centers, the majority of enzyme substrates are anionic,[1c] and transmembrane anion transport is critical for maintaining cellular functions.[2] Anion receptors therefore have potential for various biological effects. There is particular interest in promoting anion transport, thus replacing the action of defective channels which underlie genetic conditions such as cystic fibrosis.[3]

A common approach to the design of anion receptors is the deployment of hydrogen-bond-donor groups, often on scaffolds which allow multiple cooperative interactions.[4] The steroidal framework has proved highly effective, especially in the cholapods **1** 
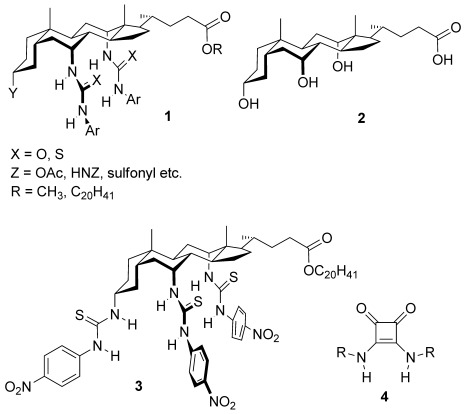
derived from cholic acid (**2**).[5] This design provides powerful preorganized binding sites embedded in lipophilic frameworks compatible with nonpolar media. The combination has yielded record affinities for anions and exceptional activities for anion transport.[6]

The strongest cholapod receptor thus far has been the tris(thiourea) **3**, which binds Et_4_N^+^Cl^−^ in chloroform with *K*_a_=2×10^11^ m^−1^.[7] Although thioureas are highly effective anion-binding functional groups,[4b] there is one alternative which is known to be more powerful. The squaramide unit **4** is more acidic, therefore a stronger hydrogen-bond donor, and also features converging NH groups.[8] This combination results in an excellent binding geometry for spherical anions (e.g. halides), or for oxygen atoms in oxoanions.[9] Simple squaramides bind halides considerably more strongly than ureas or thioureas,[9a,b, 10] and are also more active as transporters.[10] Herein we show that squaramide units placed in pairs on the rigid steroid scaffold can create outstandingly powerful receptors for anions in nonpolar solvents. Binding constants in chloroform rise to greater than 10^14^ m^−1^, the highest reported for anion recognition by electroneutral synthetic receptors. We also show that these extreme affinities do not lead to high transport activities, thus setting an important benchmark for anionophore design.

The receptors studied in this work are shown in Figure 1. N′-arylsquaramide units were placed at positions 7α and 12α of the steroid such that four NH groups converge on the central binding site. As for earlier cholapods,[5] the axial disposition of the 7,12αC—N bonds helps to preorganize the receptor. Rotation about these bonds is restricted by clashes with the steroidal framework so that the NH groups are constrained to point inwards. The electron-deficient aryl groups 4-(trifluoromethyl)phenyl and 3,5-bis-(trifluoromethyl)phenyl were employed to enhance hydrogen-bond-donor capability. Phenyl and 4-methoxyphenyl were used for comparison purposes. The steroidal 3α-position was occupied by OAc in **6**–**9** and by NHCOCF_3_ in **10** and **11**; the trifluoroacetamides are less accessible but incorporate a fifth hydrogen-bond-donor group to further enhance binding. Eicosyl ester side-chains were employed to ensure solubility in nonpolar media.[11] Calculations confirmed that the binding site should be complementary to anions such as chloride. Figure 2 shows the result of an ab initio minimization employing the model receptor **12**. The squaramide NH⋅⋅⋅Cl^−^ distances are as expected from crystallography,[12] while the structure shows no apparent strain. The formamide NH⋅⋅⋅Cl^−^ is longer than ideal, but presumably contributes to binding.

**Figure 1:**
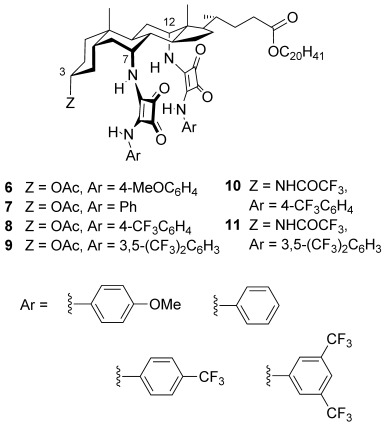
Bis(squaramid)ocholapod anion receptors studied in this work.

**Figure 2:**
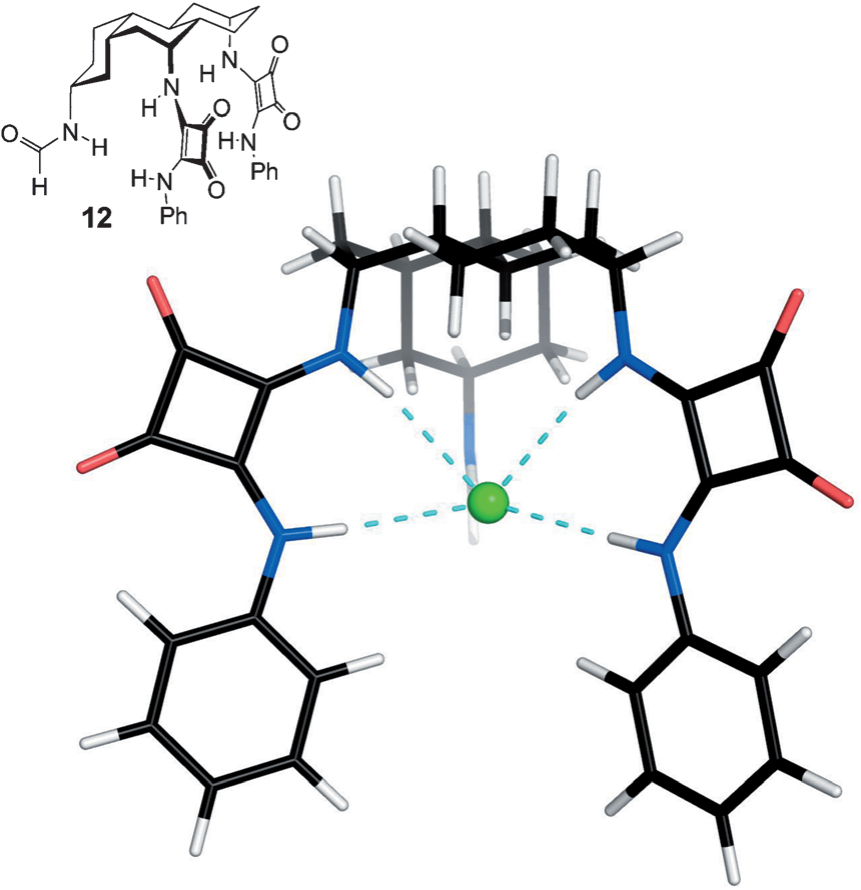
Ab initio calculated ground-state structure of 12⋅Cl^−^ (Hartree–Fock, 6-31+G* basis set). Squaramide NH⋅⋅⋅Cl^−^ distances are 2.50–2.58 Å, formamide NH⋅⋅⋅Cl^−^=3.11 Å.

The receptors **6**–**11** were prepared from the previously reported intermediates **13**[13] and **14**[14] as shown in Scheme 1.[15] The reagents for installing the squaramide units, **15**–**18**, were obtained from dimethyl squarate and aromatic amines using literature procedures.[16] Treatment of the steroidal 7α,12α-diamines with these squaramates in methanol/*i*Pr_2_NEt gave **6**–**11** in moderate to good yields (33–82 %). The corresponding ethyl squaramates proved less reactive[17] and unable to derivatize these hindered amino groups.

**Scheme 1:**
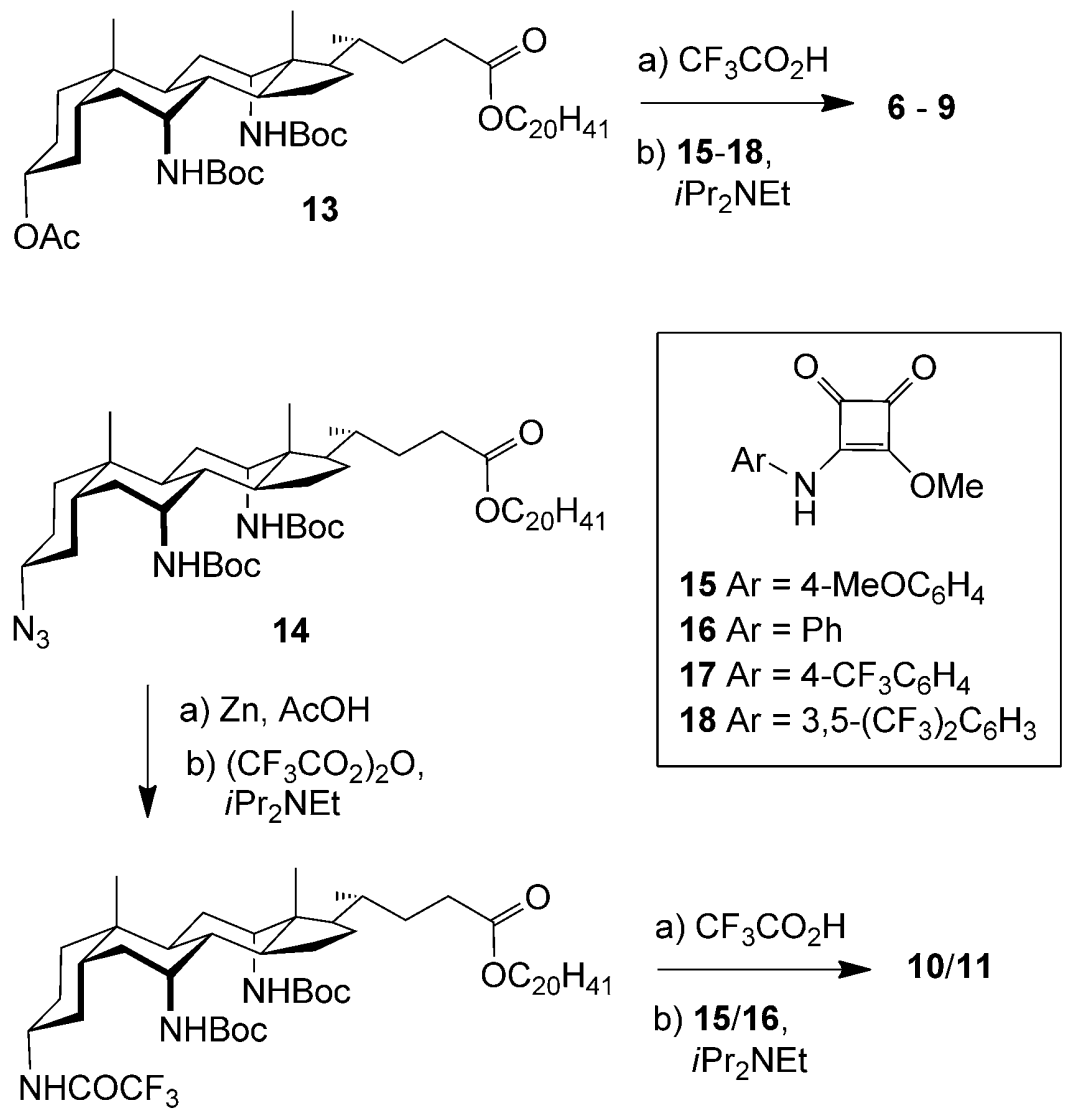
Synthesis of the receptors 6–11.

The binding properties of **6**–**11** were studied in chloroform, in line with previous work on cholapod anion receptors.[5–6] The signals in the ^1^H NMR spectra of the receptors in CDCl_3_ were broadened, but addition of increasing concentrations of R_4_N^+^Cl^−^ caused peaks to sharpen and move. Where peaks could be followed, the movements were linear with [Cl^−^] and ceased after addition of 1 equivalent. These results were consistent with 1:1 binding at affinities which (as expected) were too high for measurement by ^1^H NMR titration. To quantify binding, we employed our previously described implementation of Cram’s extraction method.[7,18,19] Briefly, the receptor is dissolved in chloroform then equilibrated with an aqueous solution of Et_4_N^+^X^−^, where X^−^ is the anion of interest. The phases are separated and the organic phase analyzed by ^1^H NMR spectroscopy to determine the amount of substrate extracted. This is used to calculate an extraction constant, *K*_e_, for the equilibrium across the phase boundary. Provided one knows the distribution constant *K*_d_ for Et_4_N^+^X^−^ between water and chloroform in the absence of receptor, the association constant *K*_a_ may be calculated as *K*_e_/*K*_d_.[15] *K*_d_ values for a number of tetraethylammonium salts between chloroform and water have been measured in previous work.[7] The analysis is subject to a number of uncertainties. For example, the receptor may aggregate in the organic phase,[20] thereby depressing the level of extraction and leading to an underestimate of *K*_a_. For these reasons, the *K*_a_ values presented in this paper should be considered “apparent”.

A particular advantage of the extraction method is its ability to measure a wide range of affinities. Quantitative complex formation must be avoided, but this can be achieved by reducing the concentration of substrate in the aqueous phase. In the present work, however, the technique was stretched to the limit. In preliminary studies, it proved difficult to avoid extracting approximately 1 equivalent of tetraethylammonium salt. The problem was only solved by using large volumes of very dilute solutions of both receptor and substrate.[15] The *K*_a_ values obtained for **6**–**11** binding Et_4_N^+^Cl^−^ in water-saturated chloroform are summarized in Table 1. Figures for the corresponding ureas **19**–**24**, 
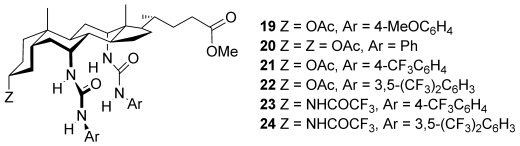
measured previously, are also given. The results in Table 1 show that, as expected, the bis(squaramido)cholapods are outstandingly powerful anion receptors. Affinities are four to five orders of magnitude higher than the corresponding ureas, rising to about 10^14^ m^−1^ for the most powerful system (**11**). As far as we are aware, these are the highest association constants reported for chloride binding to electroneutral anion receptors. Variations in binding strength follow principles established in previous work. *K*_a_ values correlate with the electron-withdrawing nature of the terminal aryl groups, and the NHCOCF_3_ unit in **10** and **11** makes an additional contribution to binding.

**Table 1:** Binding and transport data for cholapod receptors.

		Squaramides	Ureas				
Z	Ar	Compound	*K*_a_ (Et_4_N^+^Cl^−)^ [m^−1^][a]	*t*_1/2_ (Cl^−^/NO_3_^−^) [s][b]	Compound	*K*_a_ (Et_4_N^+^Cl^−)^ [m^−1^][a]	*t*_1/2_ (Cl^−^/NO_3_^−^) [s][b,c]
OAc	4-MeOC_6_H_4_	**6**	4.8×10^10^	360	**19**[21]	3.4×10^6^	n.d.
OAc	Ph	**7**	1.6×10^11^	120	**20**[21,22]	1.5×10^7^	630
OAc	4-CF_3_C_6_H_4_	**8**	2.9×10^13^	140	**21**[23]	1.8×10^8^	130
OAc	3,5-(CF_3_)_2_C_6_H_3_	**9**	4.5×10^13^	130	**22**[23]	7.7×10^8^	14
NHCOCF_3_	4-CF_3_C_6_H_4_	**10**	4.0×10^13^	150	**23**[6b]	2.5×10^9^	18
NHCOCF_3_	3,5-(CF_3_)_2_C_6_H_3_	**11**	1.2×10^14^	110	**24**[6b]	4.5×10^9^	7

[a] Apparent binding constants in water-saturated CHCl_3_. Obtained by extraction of Et_4_N^+^Cl^−^ from water into chloroform at 303 K.

[b] Half-lives for chloride/nitrate exchange in vesicles having a diameter of 200 nm. For further details see text.

[c] Recalculated from previously published data.[15]

The most powerful receptor **11** was also tested against several other monovalent anion salts, giving the results summarized in Table 2. Selectivities normalized to Et_4_N^+^Cl^−^ are also shown. The pattern is fairly similar to other cholapods with five hydrogen-bond-donor groups.[7] However, in agreement with the “affinity-selectivity principle”,[7] the differences between anions are enhanced. For example the cholapod **1** (X=O, Y=NHCOCF_3_, Ar=*p*-nitrophenyl, R=Me) was previously shown to bind Et_4_N^+^Cl^−^ with *K*_a_=1.2×10^10^ m^−1^, and Cl^−^:Br^−^:I^−^ selectivity of 1:0.5:0.1. The higher selectivity of **11** could be due to binding-site geometry, but may also result from the stronger hydrogen bonds which amplify the differences between anions.

**Table 2:** Association constants of 11 to tetraethylammonium salts in water-saturated chloroform.

Anion	*K*_a_ [m^−1^][a]	Selectivity[b]	anion	*K*_a_ [m^−1^][a]	Selectivity[b]
Cl^−^	1.2×10^14^	1	AcO^−^	3.5×10^14^	2.9
Br^−^	1.6×10^13^	0.13	ClO_4_^−^	2.5×10^10^	0.0002
I^−^	3.9×10^11^	0.003	EtSO_3_^−^	1.9×10^13^	0.16
NO_3_^−^	1.5×10^13^	0.13			

[a] Obtained by extraction of Et_4_N^+^X^−^ from water into chloroform at 303 K.

[b] Relative to Et_4_N^+^Cl^−^.

As mentioned earlier, cholapod anion receptors can also serve as anion carriers, in some cases showing remarkable activities. There is much interest in determining structure–activity relationships for anionophores, so that performance can be optimized. Anion affinity is an important parameter, and we were interested in determining how the exceptional binding power of **6**–**11** would affect their transport properties. Anion transport by the squaramides was investigated using the previously reported “lucigenin assay” for chloride/nitrate exchange in large unilamellar vesicles (LUVs).[24] Vesicles having an average diameter of 200 nm were prepared from 1-palmitoyl-2-oleoylphosphatidylcholine (POPC) and cholesterol (7:3), with transporter incorporated at a receptor/lipid ratio of 1:2500. The vesicles were prepared with internal and external aqueous NaNO_3_ (225 mm) and internal lucigenin (0.8 mm). The vesicle suspensions were placed in a fluorescence spectrometer and an external pulse of sodium chloride (25 mm) was added. The influx of Cl^−^ was followed through the decay in lucigenin fluorescence (Figure 3). The transport rates were quantified through fitting to a single exponential decay function to give approximate half-lives (*t*_1/2_, s).

**Figure 3:**
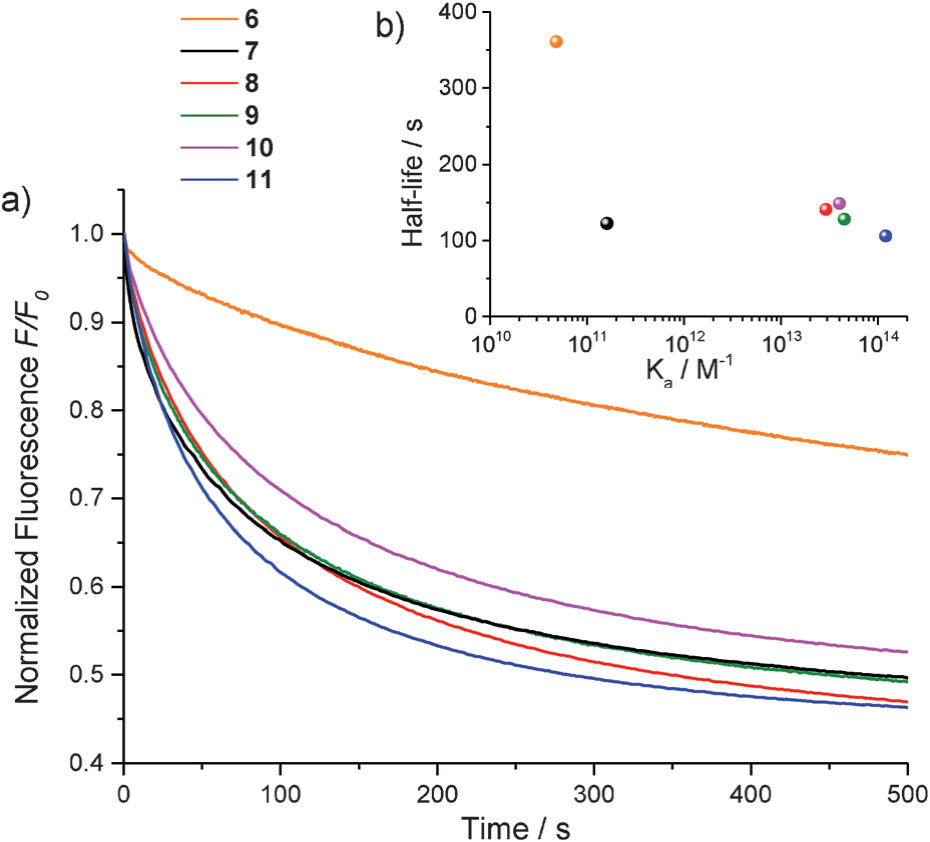
a) Chloride/nitrate exchange by 6–11 at receptor/lipid=1:2500 and detected by the lucigenin method (see text). b) Plot of transport half-lives versus *K*_a_ (Et_4_N^+^Cl^−^, CHCl_3_) for 6–11.

The half-lives for **6**–**11** are listed in Table 1 along with the values for **20**–**24**. Interestingly, the variation across each series is very different. For **20**–**24**, raising the affinities yields dramatic improvements in transport rates. The correlation is not perfect, but the general trend is clear. In contrast, for **6**–**11**, the same trend applies at the beginning of the series (**6**→**7**) but transport rates then plateau (as is directly apparent from Figure 3). Thus while **7** is five times more effective than the corresponding bis(urea) **20**, the squaramides lose this advantage as affinities increase further. Although none of the bis(squaramide)s possess exceptional transport activity, the results may provide mechanistic insight. It is reasonable to assume that the moderate performance of **7**–**11** is linked to their exceptional affinities.[25] It is well understood that as binding strength increases, a point is reached where further enhancements are unproductive.[26,27] However, one would normally expect that transport rates would start falling with increasing affinities, as anion release becomes rate determining. A possible explanation is that the very strong receptors do not decomplex, but undergo direct anion exchange at the membrane surface. The exchange could involve one substrate for the other (e.g. chloride for nitrate), or substrate for a phospholipid head group. In either case the rate might not be very sensitive to affinity, as the transition state would require simultaneous formation and cleavage of hydrogen bonds. The hypothesis implies that powerful receptors should perform relatively poorly in tests involving cation–anion co-transport (for example, salt transport assisted by cation carriers). Preliminary experiments suggest that this is indeed the case for the bis(squaramide)s.[28] If confirmed, this could add complexity to the design of anion carriers. It may be necessary to distinguish between different mechanisms (unidirectional anion transport and exchange) and to apply different design criteria depending on the activity required.

In conclusion we have found that steroid-based anion receptors with axial squaramide units are capable of extreme affinities for tetra-alkylammonium salts in chloroform. The affinities are achieved without employing electrostatic interactions or Lewis-acidic metals, and are testament to the effectiveness of hydrogen bonding when carefully deployed. The transport properties of these molecules suggest that finally a limit has been reached beyond which affinity and anionophore activity no longer correlate. This information, and the unexpected shape of the affinity-activity plot, will provide valuable guidance for future anionophore design.
