# Supplementary material for: High-Affinity Anion Binding by Steroidal Squaramide Receptors
Source: Angew Chem Int Ed Engl. 2015 Feb 17;54(15):4592–6. doi: 10.1002/anie.201411805 (PMC4405043; doi:10.1002/anie.201411805)
Supplement: Supplementary file 1 [file anie0054-4592-sd1.pdf]

Angewandte Chemie
Eine Zeitschrift der Gesellschaft Deutscher Chemiker

Supporting Information

High-Affinity Anion Binding by Steroidal Squaramide Receptors**

Sophie J. Edwards, Hennie Valkenier, Nathalie Busschaert, Philip A. Gale, and Anthony P. Davis**

anie_201411805_sm_miscellaneous_information.pdf

Supporting Information

Contents

1. Synthesis 2
General methods 2
Synthesis of new squaramide receptors 3
2. Binding studies 19
Preparation of $Et_4N^+EtSO_3^-$ by neutralisation 19
General procedure for extraction studies in chloroform by Cram's extraction method 19
K_a values for chloride anion binding by extraction method 21
K_a values for binding to various anions by extraction method 22
^{1}H NMR titration studies 23
^{1}H NMR titration of receptor **10** with $Bu_4N^+Cl^-$ in $CDCl_3$ 24
^{1}H NMR titration of receptor **11** with $Bu_4N^+Cl^-$ in $CDCl_3$ 25
3. Transport Studies 26
General procedure for transport measurements 26
Chloride transport into vesicles by eicosyl ester squaramides **6-11** 28
References and Notes 29

1. Synthesis

General methods

All reagents were purchased from commercial suppliers and used without further purification, unless otherwise stated. Anhydrous DCM was dried by passing through a modified Grubbs system, with an alumina column manufactured by Anhydrous Engineering. Anhydrous MeOH was purchased from a commercial supplier and used as received.

Flash column chromatography was performed using silica gel (Fisher brand silica 60 Å particle size 35-70 micron) as the absorbent. Routine monitoring of reactions was performed using precoated silica gel TLC plates (Merck silica gel 60 F_{254}). Spots were visualised under UV light or by staining with phosphomolybdic acid, potassium permanganate, or ninhydrin; R_f values are given under these conditions.

^{1}H, ^{13}C and ^{19}F NMR spectra were recorded using a ECP 300, ECP 400, Varian 400, Varian System 500A (carbon sensitive probe) or Varian System 500B (proton sensitive probe) spectrometer. All spectra were recorded at 298 K unless otherwise stated. Chemical shifts (δ) are quoted in parts per million (ppm), coupling constants (*J*) are quoted in Hz and spectra are referenced to the appropriate residual solvent peak. NMR spectra for characterisation of the bis-squaramide receptors were obtained in $CDCl_3$ in the presence of two equivalents of $Et_4N^+Cl^-$, conditions which gave well-resolved and reproducible spectra. Mass spectra were recorded on a Bruker microTOF II (ESI), VG Analytical Quattro (ESI) or VG Analytical Autospec (EI). IR spectra were recorded on a Perkin-Elmer Spectrum 100 FT-IR spectrometer. Elemental analysis was carried out by the microanalysis department at the School of Chemistry, University of Bristol.

The carbon numbering system for steroids is as below:

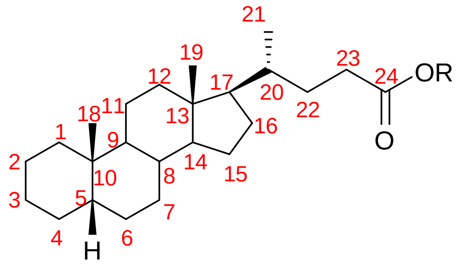

Synthesis of new squaramide receptors

The six new squaramides described in this communication were synthesised as outlined in Scheme S1 below, starting from previously reported intermediate **A**.[1] Intermediates **B**, **C**, and **13** were prepared from intermediate **A** as previously described.[1,2] Known intermediate **14**[3] was prepared according to a modified literature procedure and novel compound **D** was prepared from intermediate **14**. The squaramates **15-18** were prepared from squaric acid according to literature procedures.[4]

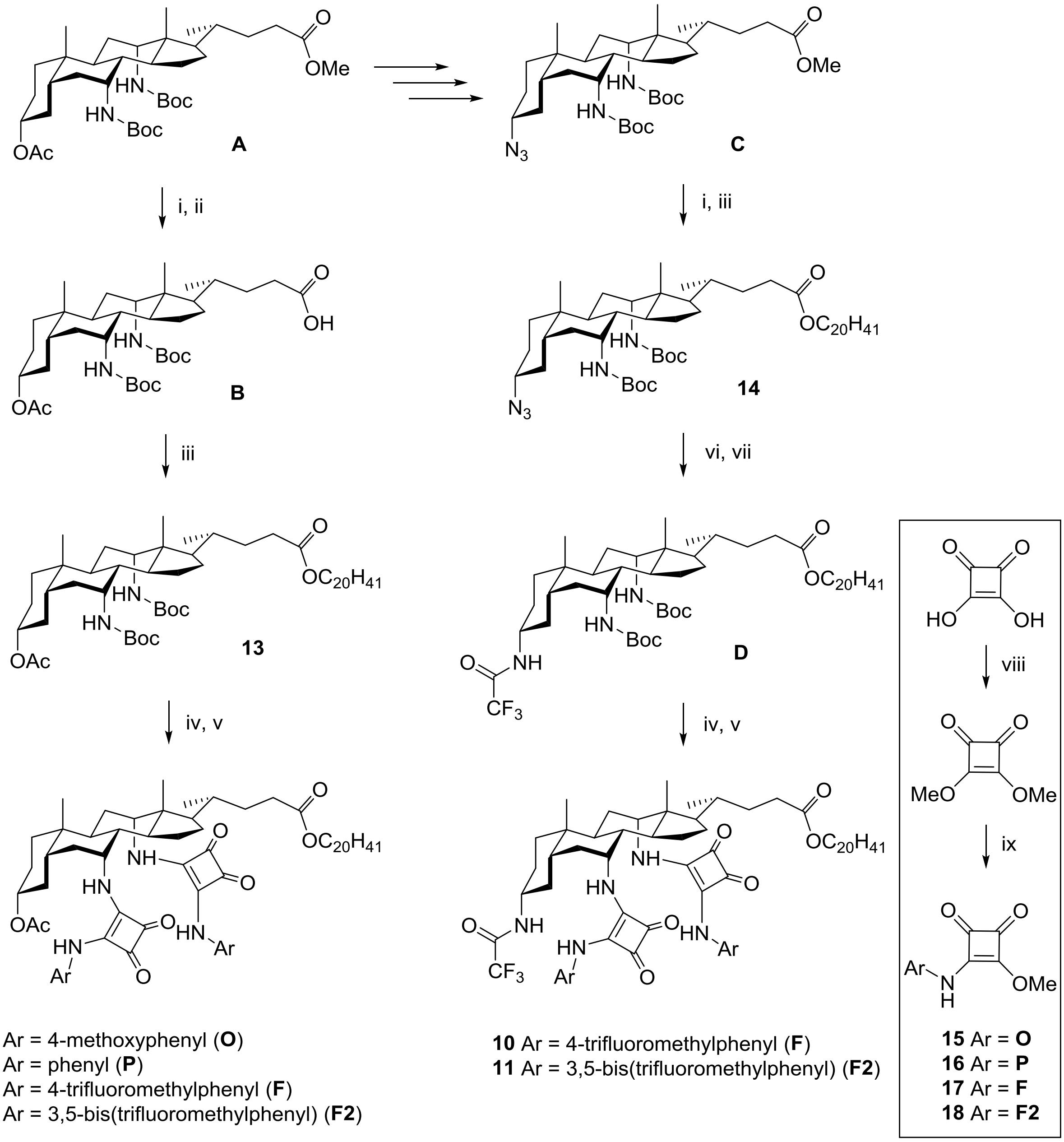

Scheme S1. (i) KOH, EtOH/H_2O, rt, 24 h; (ii) pyridine, Ac_2O, rt, 24 h; (iii) $CH_3(CH_2)_{19}OH$, DMAP, EDCI, DCM, rt, 16 h; (iv) TFA, DCM, rt, 16 h (v) **15-18**, DIPEA, MeOH, rt (**6** at 50°C), 24-48 h; (vi) Zn, AcOH, rt, 16 h; (vii) TFAA, DIPEA, DCM, rt, 3h; (viii) $CH(OMe)_3$, MeOH, reflux, 24 h; (ix) Ar-NH_2, MeOH, rt, 6-48 h.

Cholapod receptor 6

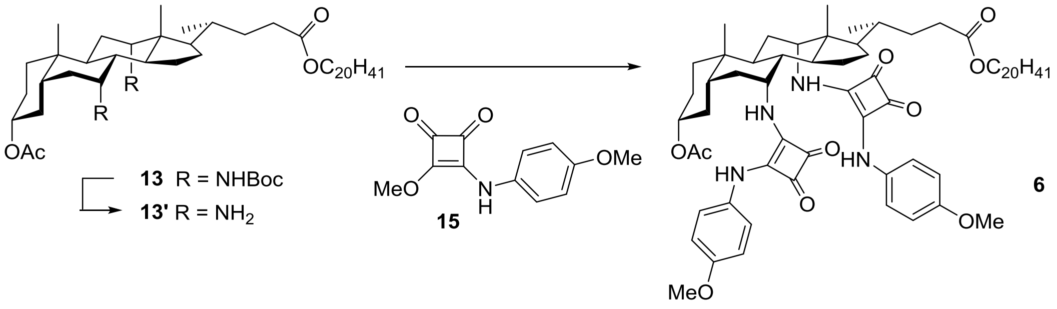

To a solution of Boc-protected diamine **13** (95 mg, 0.10 mmol) in anhydrous DCM (1.6 mL) was added TFA (1.4 mL) dropwise over 5 minutes. The solution was stirred at room temperature, under N_2, for 16 hours. The solvent was removed *in vacuo* and the residue redissolved in DCM (40 mL) and washed with saturated aqueous $NaHCO_3$ (2 × 20 mL). The aqueous phases were extracted with DCM (2 × 20 mL) and the combined organic extracts were dried (Na_2SO_4), filtered and concentrated *in vacuo* yielding the crude diamine **13'** (69 mg, 93%).

To a solution of diamine **13'** (69 mg, 0.10 mmol) in anhydrous MeOH (1 mL) was added squaramate **15** (130 mg, 0.560 mmol) and DIPEA (0.10 mL, 0.57 mmol). The resulting suspension was stirred at 50 °C, under N_2, for 48 hours. The solvent was removed *in vacuo* and the crude residue was purified by flash column chromatography (5% MeOH in DCM). The obtained yellow material was dissolved in EtOAc (40 mL), washed with aqueous H_2SO_4 (20 mL, 0.5 M)[5] and water (15 mL), dried ($MgSO_4$), filtered and concentrated *in vacuo* yielding squaramide receptor **6** (60 mg, 56%) as a yellow solid: R_f 0.2 (10% MeOH in DCM); ν_{max} (neat)/cm^{-1} 3252 (NH), 2923 (CH), 2852 (CH), 1792 (C=O), 1731 (C=O), 1677, 1603, 1574, 1526, 1502, 1438, 1364, 1241, 1173, 1081, 1027, 897, 751, 689, 661; HRMS (ESI) calc. for $[C_{68}H_{98}N_4O_{10}Na]^+$ 1153.7175, found 1153.7179; elemental analysis $[C_{68}H_{98}N_4O_{10} + 2.H_2O]$ calculated C 69.95, H 8.81, N 4.80, found C 70.25, H 8.80, N 4.80; NMR spectra were obtained on a solution of **6** (2 mM) in $CDCl_3$ to which $Et_4N^+Cl^-$ (2 eq) had been added: ^{1}H NMR (500 MHz, $CDCl_3$) δ 0.84 (3H, s, 18-H_3), 0.88 (3H, t, *J* 6.8, CH_2CH_3), 0.91 (3H, d, *J* 6.7, 21-H_3), 0.96 (3H, s, 19-H_3), 1.22-1.28 (34H, br m, $OCH_2CH_2(CH_2)_{17}CH_3$), 1.37 (12H, t, *J* 7.3, Et_4N^+ CH_3), 1.95 (3H, s, CH_3CO_2), 2.26-2.35 (1H, m), 2.39-2.49 (1H, m), 3.41 (q, *J* 7.3, Et_4N^+ CH_2), 3.79 (6H, s, 2 × OCH_3), 4.00 (2H, t, *J* 6.7, $CO_2CH_2CH_2(CH_2)_{17}CH_3$), 4.23 (1H, br s, 7β-H), 4.45-4.54 (1H, m, 3β-H), 4.72-4.79 (1H, m, 12β-H), 6.70 (2H, d, *J* 8.9, 2 × ArH), 6.88 (2H, d, *J* 8.9, 2 × ArH), 7.67 (2H, d, *J* 8.9, 2 × ArH), 7.68 (2H, d, *J* 8.9, 2 × ArH), 8.42 (1H, d, *J* 10.9, 12α-NH), 8.47 (1H, d, *J* 7.7, 7α-NH), 10.28 (1H, s, C-NH), 10.33 (1H, s, C-NH); ^{13}C NMR (126 MHz, $CDCl_3$) δ 8.0 (Et_4N^+ CH_3), 13.3 (18-CH_3), 14.3 (CH_2CH_3), 18.4 (21-CH_3), 21.8 (CH_3CO_2), 22.7 (19-CH_3), 22.8 (CH_2),

23.3 (CH_2), 26.0 (CH_2), 26.8 (CH_2), 26.9 (CH_2), 28.3 (CH), 28.8 (CH_2), 28.9 (CH_2), 29.4 (CH_2), 29.5 (CH_2), 29.7 (CH_2), 29.7 (CH_2), 29.8 (CH_2), 29.8 (CH_2), 30.7 (CH_2), 31.8 (CH_2), 32.1, 34.5, 34.7, 34.8, 35.0, 35.1, 37.5 (CH), 41.5 (CH), 43.2 (CH), 45.7 (C), 48.8 (CH), 52.3 (7-CH), 53.0 (Et_4N^+ CH_2), 55.7 (ArCO*C*H_3), 57.9 (12-CH), 64.6 (O*C*$H_2(CH_2)_{18}CH_3$), 74.5 (3-CH), 114.5 (ArCH), 120.1 (ArCH), 130.3 (ArCNH), 130.4 (ArCNH), 155.6 (Ar*C*OCH_3), 155.7 (Ar*C*OCH_3), 165.2 (*C*=C), 165.3 (*C*=C), 168.5 (C=*C*), 169.2 (C=*C*), 171.3 (CH_3*C*O_2), 174.4 (*C*$O_2CH_2(CH_2)_{18}CH_3$), 180.7 (CO), 181.1 (CO), 183.3 (CO), 183.4 (CO).

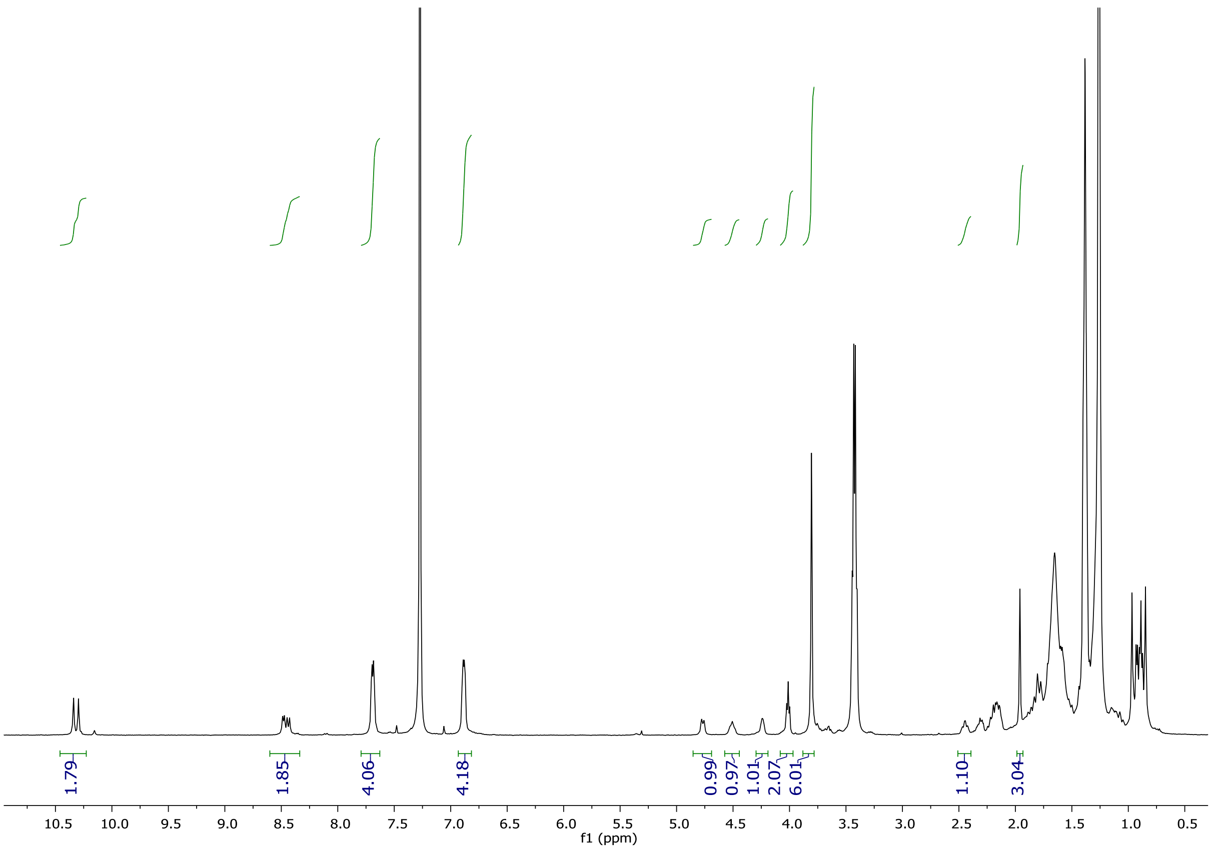

Figure S1. ^1H NMR spectrum of receptor **6** in $CDCl_3$ in the presence of 2 equivalents of $Et_4N^+Cl^-$.

Cholapod receptor 7

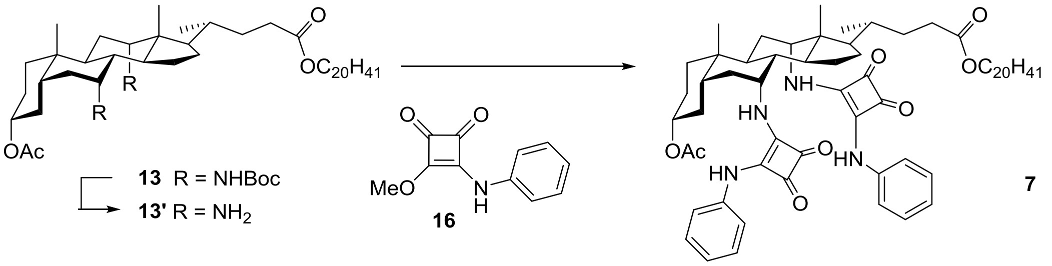

To a solution of Boc-protected diamine **13** (95 mg, 0.10 mmol) in anhydrous DCM (1.6 mL) was added TFA (1.4 mL) dropwise over 5 minutes. The solution was stirred at room temperature, under N_2, for 16 hours. The solvent was removed *in vacuo* and the residue redissolved in DCM (40 mL) and washed with saturated aqueous $NaHCO_3$ (2 × 20 mL). The aqueous phases were extracted with DCM (2 × 20 mL) and the combined organic extracts were dried (Na_2SO_4), filtered and concentrated *in vacuo* yielding the crude diamine **13'** (74 mg, quant.).

To a solution of diamine **13'** (74 mg, 0.10 mmol) in anhydrous MeOH (1 mL) was added squaramate **16** (63 mg, 0.31 mmol) and DIPEA (50 µL, 0.29 mmol). The resulting suspension was stirred at room temperature, under N_2, for 48 hours. The solvent was removed *in vacuo* and the crude residue was purified by flash column chromatography (5% MeOH in DCM). The obtained yellow material was dissolved in EtOAc (20 mL), washed with aqueous H_2SO_4 (10 mL, 0.5 M)[5] and water (10 mL), dried ($MgSO_4$), filtered and concentrated *in vacuo* yielding squaramide receptor **7** (36 mg, 33%) as a yellow solid: R_f 0.2 (10% MeOH in DCM); ν_{max} (neat)/cm^{-1} 3254 (NH), 2922 (CH), 2852 (CH), 1792 (C=O), 1731 (C=O), 1676, 1603, 1573, 1526, 1502, 1439, 1364, 1241, 1173, 1081, 1026, 897, 751, 689, 661; HRMS (ESI) calc. for $[C_{66}H_{94}N_4O_8Na]^+$ 1093.6951, found 1093.6964; elemental analysis $[C_{66}H_{94}N_4O_8 + 1.H_2O]$ calculated C 72.76, H 8.88, N 5.14, found C 72.71, H 8.77, N 5.11; NMR spectra were obtained on a solution of **7** (2 mM) in $CDCl_3$ to which $Et_4N^+Cl^-$ (2 eq) had been added: ^{1}H NMR (500 MHz, $CDCl_3$) δ 0.84 (3H, s, 18-H_3), 0.88 (3H, t, *J* 6.9, CH_2CH_3), 0.91 (3H, d, *J* 6.7, 21-H_3), 0.96 (3H, s, 19-H_3), 1.19-1.32 (34H, br m, $OCH_2CH_2(CH_2)_{17}CH_3$), 1.37 (12H, tt, *J* 7.3 and 1.7 Et_4N^+ CH_3), 1.94 (3H, s, CH_3CO_2), 2.30 (1H, ddd, *J* 15.0, 9.6 and 5.1), 2.46 (1H, td, *J* 12.5 and 3.8), 3.41 (q, *J* 7.3, Et_4N^+ CH_2), 3.99 (2H, t, *J* 6.8, $CO_2CH_2CH_2(CH_2)_{17}CH_3$), 4.24 (1H, br s, 7β-H), 4.45-4.54 (1H, m, 3β-H), 4.73-4.79 (1H, m, 12β-H), 7.01 (2H, t, *J* 7.4, 2 × ArH), 7.29-7.36 (4H, m, 4 × ArH), 7.79 (4H, d, *J* 8.6, 4 × ArH), 8.52 (1H, d, *J* 11.0, 12α-NH), 8.56 (1H, d, *J* 7.8, 7α-NH), 10.41 (1H, s, C-NH), 10.45 (1H, s, C-NH); ^{13}C NMR (126 MHz, $CDCl_3$) δ 7.9 (Et_4N^+ CH_3), 13.3 (18-CH_3), 14.3 (CH_2CH_3), 18.4 (21-CH_3), 21.7 (CH_3CO_2), 22.7 (19-CH_3), 22.8 (CH_2), 23.3 (CH_2), 26.1 (CH_2), 26.8 (CH_2), 26.9 (CH_2), 28.3 (CH), 28.8 (CH_2), 28.9 (CH_2), 29.4 (CH_2), 29.5 (CH_2), 29.7

(CH_2), 29.7 (CH_2), 29.8 (CH_2), 29.8 (CH_2), 30.7 (CH_2), 31.8 (CH_2), 32.1, 34.5, 34.7, 34.8, 35.0, 35.1, 37.5 (CH), 41.5 (CH), 43.3 (CH), 45.8 (C), 48.9 (CH), 52.4 (7-CH), 52.9 (Et_4N^+ CH_2), 57.9 (12-CH), 64.6 ($O\mathit{C}H_2(CH_2)_{18}CH_3$), 74.5 (3-CH), 118.7 (ArCH), 122.7 (ArCH), 122.8 (ArCH), 129.2 (ArCH), 129.3 (ArCH), 139.9 (ArCNH), 139.9 (ArCNH), 165.4 (*C*=C), 165.4 (*C*=C), 168.8 (C=*C*), 169.6 (C=*C*), 171.3 ($CH_3\mathit{C}O_2$), 174.4 ($\mathit{C}O_2CH_2(CH_2)_{18}CH_3$), 180.6 (CO), 181.0 (CO), 183.9 (CO), 184.1 (CO).

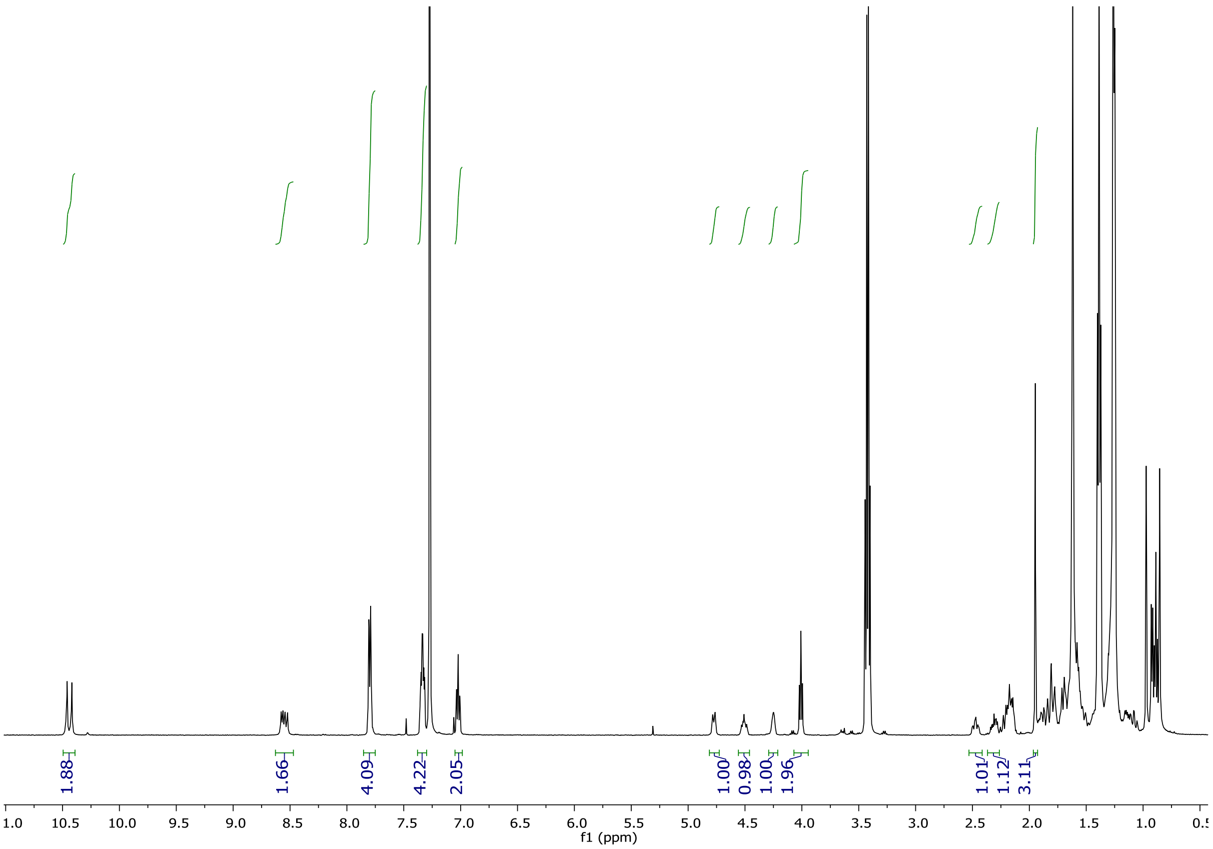

Figure S2. ^1H NMR spectrum of receptor **7** in $CDCl_3$ in the presence of 2 equivalents of $Et_4N^+Cl^-$.

Cholapod receptor 8

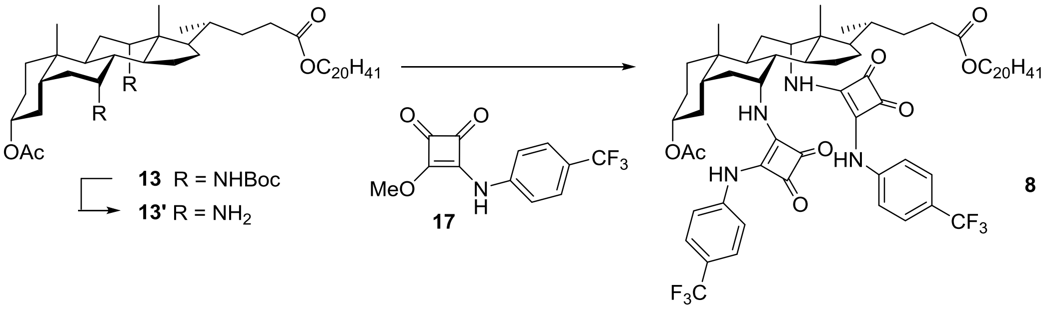

To a solution of Boc-protected diamine **13** (190 mg, 0.204 mmol) in anhydrous DCM (2.8 mL) was added TFA (2.6 mL) dropwise over 5 minutes. The solution was stirred at room temperature, under N_2, for 16 hours. The solvent was removed *in vacuo* and the residue redissolved in DCM (50 mL) and washed with saturated aqueous $NaHCO_3$ (2 × 30 mL). The aqueous phases were extracted with DCM (2 × 30 mL) and the combined organic extracts were dried (Na_2SO_4), filtered and concentrated *in vacuo* yielding the crude diamine **13'** (135 mg, 91%).

To a solution of diamine **13'** (135 mg, 0.185 mmol) in anhydrous MeOH (1 mL) was added squaramate **17** (150 mg, 0.553 mmol) and DIPEA (0.10 mL, 0.57 mmol). The resulting suspension was stirred at room temperature, under N_2, for 24 hours. The solvent was removed *in vacuo* and the crude residue was purified by flash column chromatography (5% MeOH in DCM). The obtained yellow material was dissolved in EtOAc (40 mL), washed with aqueous H_2SO_4 (20 mL, 0.5 M)[5] and water (2 × 20 mL), dried ($MgSO_4$), filtered and concentrated *in vacuo* yielding squaramide receptor **8** (134 mg, 60%) as a yellow solid: R_f 0.2 (10% MeOH in DCM); ν_{max} (neat)/cm^{-1} 3445 (NH), 3174 (NH), 2952 (CH), 2872 (CH), 1794 (C=O), 1719 (C=O), 1677, 1612, 1581, 1535, 1443, 1421, 1380, 1317, 1266, 1188, 1162, 1113, 1067, 1028, 1016, 840; HRMS (ESI) calc. for $[C_{68}H_{92}F_6N_4O_8Na]^+$ 1229.6723, found 1229.6712; elemental analysis [$C_{68}H_{92}F_6N_4O_8$ + 1.H_2O] calculated C 66.65, H 7.73, N 4.57, found C 66.20, H 7.50, N 4.71; NMR spectra were obtained on a solution of **8** (2 mM) in $CDCl_3$ to which $Et_4N^+Cl^-$ (2 eq) had been added: ^{1}H NMR (500 MHz, $CDCl_3$) δ 0.85 (3H, s, 18-H_3), 0.88 (3H, t, *J* 6.9, CH_2CH_3), 0.91 (3H, d, *J* 6.5, 21-H_3), 0.96 (3H, s, 19-H_3), 1.19-1.31 (34H, br m, $OCH_2CH_2(CH_2)_{17}CH_3$), 1.39 (12H, tt, *J* 7.3 and 1.7, Et_4N^+ CH_3), 1.93 (3H, s, CH_3CO_2), 2.30 (1H, ddd, *J* 15.0, 9.5 and 5.1), 2.42 (1H, td, *J* 12.4 and 4.1), 3.44 (q, *J* 7.3, Et_4N^+ CH_2), 4.00 (2H, t, *J* 6.8, $CO_2CH_2CH_2(CH_2)_{17}CH_3$), 4.24 (1H, br s, 7β-H), 4.46-4.54 (1H, m, 3β-H), 4.74-4.80 (1H, m, 12β-H), 7.57 (4H, d, *J* 8.6, 4 × ArH), 7.88 (4H, d, *J* 8.6, 4 × ArH), 8.48 (1H, d, *J* 10.9, 12α-NH), 8.53 (1H, d, *J* 8.0, 7α-NH), 10.52 (1H, s, C-NH), 10.56 (1H, s, C-NH); ^{13}C NMR (126 MHz, $CDCl_3$) δ 7.9 (Et_4N^+ CH_3), 13.3 (18-CH_3), 14.3 (CH_2CH_3), 18.3 (21-CH_3), 21.7 (CH_3CO_2), 22.6 (19-CH_3), 22.8 (CH_2),

23.2 (CH_2), 26.0 (CH_2), 26.8 (CH_2), 26.9 (CH_2), 28.3 (CH), 28.8 (CH_2), 28.9 (CH_2), 29.4 (CH_2), 29.5 (CH_2), 29.6 (CH_2), 29.7 (CH_2), 29.8 (CH_2), 29.8 (CH_2), 30.7 (CH_2), 31.7 (CH_2), 32.1, 34.5, 34.7, 34.8, 34.9, 35.0, 37.5 (CH), 41.4 (CH), 43.3 (CH), 45.7 (C), 48.9 (CH), 52.5 (7-CH), 52.9 (Et_4N^+ CH_2), 58.11 (12-CH), 64.61 ($OCH_2(CH_2)_{18}CH_3$), 74.38 (3-CH), 118.29 (ArCH), 124.1 (Ar*C*CF_3), 124.5 (q, $J_{C\text{-}F}$ 267.9, ArC*C*F_3), 126.5 (ArCH), 142.9 (ArCNH), 143.0 (ArCNH), 164.8 (C=*C*), 164.9 (C=*C*), 169.2 (C=*C*), 169.9 (C=*C*), 171.2 (CH_3CO_2), 174.3 ($CO_2CH_2(CH_2)_{18}CH_3$), 180.4 (CO), 180.8 (CO), 184.5 (CO), 184.6 (CO); ^{19}F NMR (470 MHz, $CDCl_3$) δ -61.74, -61.75.

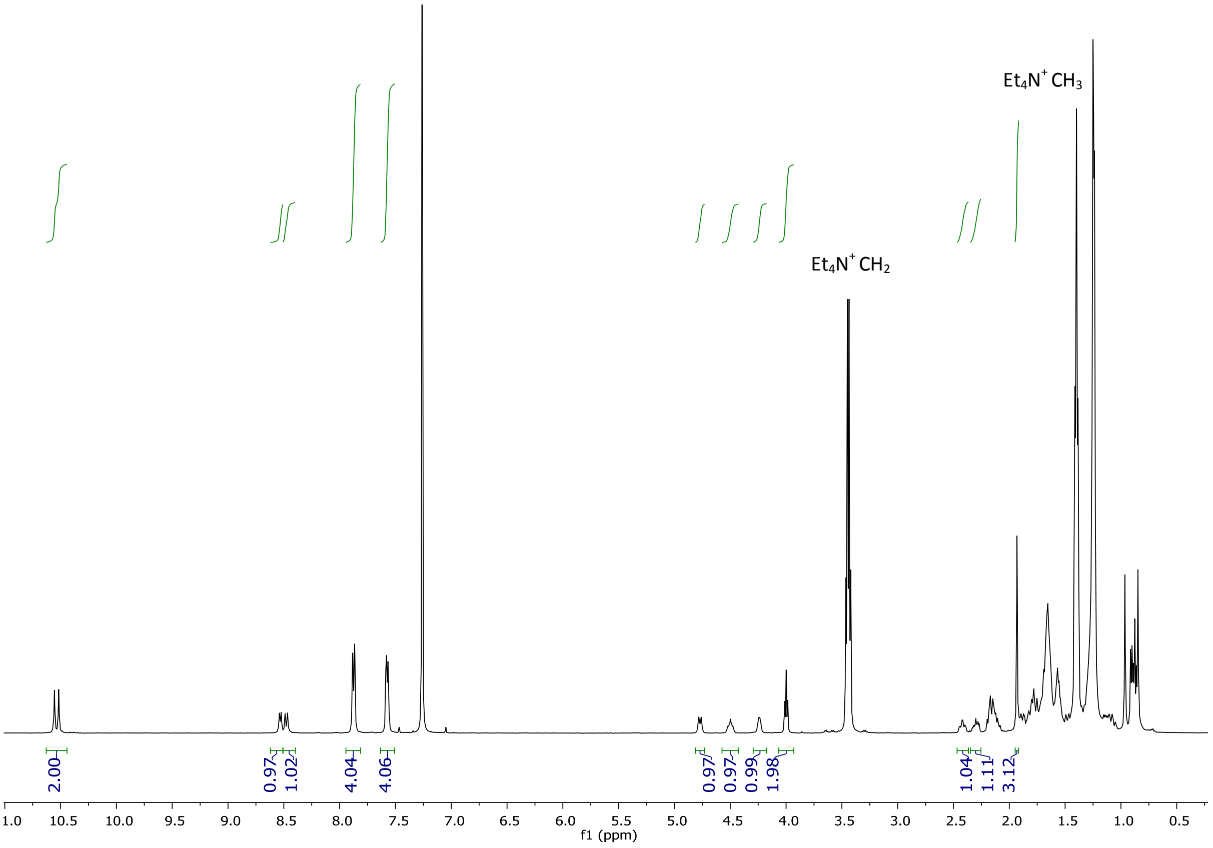

Figure S3. ^1H NMR spectrum of receptor **8** in $CDCl_3$ in the presence of 2 equivalents of $Et_4N^+Cl^-$.

Cholapod receptor 9

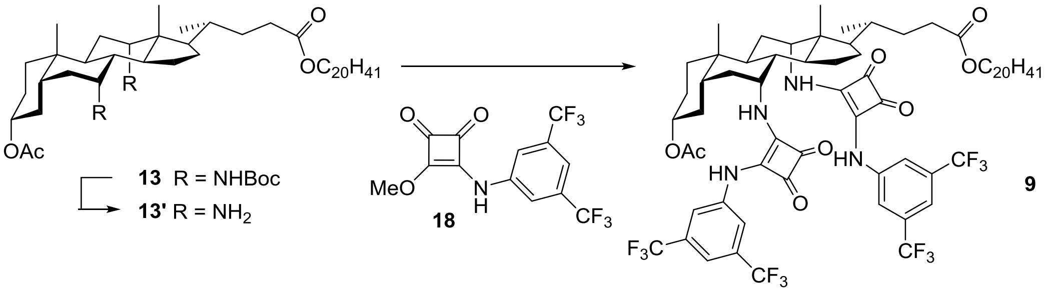

To a solution of Boc-protected diamine **13** (180 mg, 0.194 mmol) in anhydrous DCM (2.5 mL) was added TFA (2.5 mL) dropwise over 5 minutes. The solution was stirred at room temperature, under N_2, for 16 hours. The solvent was removed *in vacuo* and the residue redissolved in DCM (50 mL) and washed with saturated aqueous $NaHCO_3$ (2 × 30 mL). The aqueous phases were extracted with DCM (2 × 30 mL) and the combined organic extracts were dried (Na_2SO_4), filtered and concentrated *in vacuo* yielding the crude diamine **13'** (133 mg, 94%).

To a solution of diamine **13'** (133 mg, 0.182 mmol) in anhydrous MeOH (1 mL) was added squaramate **18** (186 mg, 0.547 mmol) and DIPEA (0.10 mL, 0.57 mmol). The resulting suspension was stirred at room temperature, under N_2, for 24 hours. The solvent was removed *in vacuo* and the crude residue was purified by flash column chromatography (5% MeOH in DCM). The obtained yellow material was dissolved in EtOAc (40 mL), washed with aqueous H_2SO_4 (20 mL, 0.5 M)[5] and water (2 × 20 mL), dried ($MgSO_4$), filtered and concentrated *in vacuo* yielding squaramide receptor **9** (162 mg, 66%) as a yellow solid: R_f 0.2 (10% MeOH in DCM); ν_{max} (neat)/cm^{-1} 3175 (NH), 2932 (CH), 2871 (CH), 1794 (C=O), 1720 (C=O), 1677, 1611, 1581, 1535, 1441, 1377, 1317, 1270, 1185, 1163, 1113, 1067, 1027, 1016, 840; HRMS (ESI) calc. for $[C_{70}H_{90}F_{12}N_4O_8Na]^+$ 1365.6453, found 1365.6459; elemental analysis $[C_{70}H_{90}F_{12}N_4O_8 + 1.H_2O]$ calculated C 61.75, H 6.81, N 4.12, found C 61.39, H 6.74, N 4.24; NMR spectra were obtained on a solution of **9** (2 mM) in $CDCl_3$ to which $Et_4N^+Cl^-$ (2 eq) had been added: ^{1}H NMR (500 MHz, $CDCl_3$) δ 0.89 (3H, s, 18-H_3), 0.88 (3H, t, *J* 7.0, CH_2CH_3), 0.92 (3H, d, *J* 6.6, 21-H_3), 0.96 (3H, s, 19-H_3), 1.21-1.31 (34H, br m, $OCH_2CH_2(CH_2)_{17}CH_3$), 1.41 (12H, tt, *J* 7.3 and 1.7, Et_4N^+ CH_3), 1.93 (3H, s, CH_3CO_2), 2.26-2.41 (2H, m), 3.46 (q, *J* 7.3, Et_4N^+ CH_2), 4.00 (2H, t, *J* 6.8, $CO_2CH_2CH_2(CH_2)_{17}CH_3$), 4.25 (1H, br s, 7β-H), 4.45-4.55 (1H, m, 3β-H), 4.76-4.84 (1H, m, 12β-H), 7.47 (2H, br s, 2 × ArH), 8.24 (2H, s, 2 × ArH), 8.26 (2H, s, 2 × ArH), 8.36 (1H, d, *J* 10.9, 12α-NH), 8.51 (1H, d, *J* 7.8, 7α-NH), 10.53 (1H, s, C-NH), 10.58 (1H, s, C-NH); ^{13}C NMR (126 MHz, $CDCl_3$) δ 7.9 (Et_4N^+ CH_3), 13.3 (18-CH_3), 14.3 (CH_2CH_3), 18.1 (21-CH_3), 21.5 (CH_3CO_2), 22.6 (19-CH_3), 22.8 (CH_2), 23.2 (CH_2), 26.0 (CH_2), 26.7 (CH_2), 27.2 (CH_2), 28.3 (CH), 28.8 (CH_2), 29.1 (CH_2), 29.4 (CH_2), 29.5 (CH_2), 29.7 (CH_2), 29.7

(CH$_2$), 29.8 (CH$_2$), 29.8 (CH$_2$), 30.8 (CH$_2$), 31.6 (CH$_2$), 32.1, 34.4, 34.6, 34.8, 34.9, 35.0, 37.3 (CH), 41.3 (CH), 43.6 (CH), 45.5 (C), 48.9 (CH), 52.5 (7-CH), 52.9 (Et_4N^+ CH$_2$), 58.3 (12-CH), 64.6 (O*C*H$_2$(CH$_2$)$_{18}$CH$_3$), 74.2 (3-CH), 115.6 (ArCH), 118.5 (ArCH), 123.4 (q, $J_{C\text{-}F}$ 272.0, ArC*C*F$_3$), 132.6 (q, $^2J_{C\text{-}F}$ 33.7, Ar*C*CF$_3$), 141.2 (ArCNH), 141.3 (ArCNH), 164.3 (*C*=C), 164.6 (*C*=C), 169.2 (C=*C*), 169.9 (C=*C*), 171.3 (CH$_3$*C*O$_2$), 174.3 (*C*O$_2$CH$_2$(CH$_2$)$_{18}$CH$_3$), 180.4 (CO), 180.9 (CO), 184.5 (CO), 184.7 (CO); ^{19}F NMR (470 MHz, CDCl$_3$) δ -63.24, -63.27.

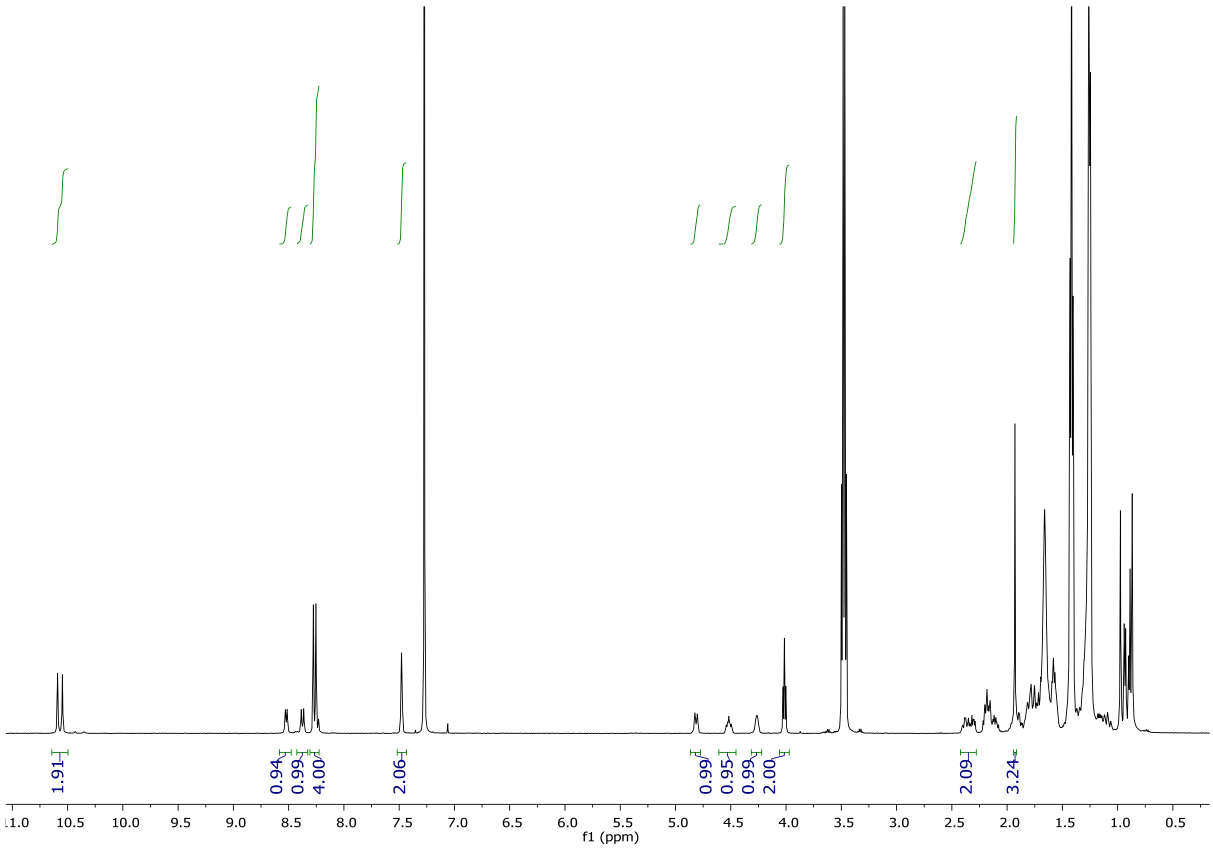

Figure S4. ^{1}H NMR spectrum of receptor **9** in CDCl$_3$ in the presence of 2 equivalents of $Et_4N^+Cl^-$.

Eicosanoyl 3α-azido-7α,12α-di[*N*-(*t*-butoxycarbonyl)amino]-5β-cholan-24-oate 14

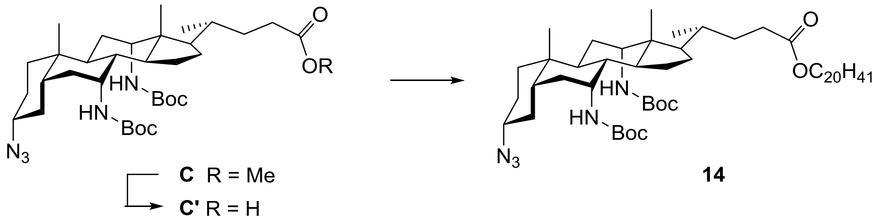

A solution of methyl ester **C** (3.86 g, 5.98 mmol) and KOH (1.31 g, 23.3 mmol) in EtOH (200 mL) and water (24 mL) was stirred at room temperature for 24 hours. The solvent was removed *in vacuo* and the residue partitioned between DCM (250 mL) and water (250 mL). Aqueous HCl (2 M) was added dropwise until the pH of the aqueous layer was ~ 2-4. The organic phase was separated, and the aqueous phase extracted with DCM (2 × 100 mL). The combined organic phases were washed with water (50 mL), dried (Na_2SO_4), filtered and concentrated *in vacuo* yielding crude acid **C'** (2.79 g, 74%) as a white solid: R_f 0.5 (50:50 EtOAc:hexane); HRMS (ESI) calc. for $[C_{34}H_{57}N_5O_6Na]^+$ 654.4201, found 654.4206.

To a solution of acid **C'** (2.78 g, 4.40 mmol), eicosanol (1.25 g, 4.18 mmol) and DMAP (1.75 g, 14.4 mmol) in anhydrous DCM (30 mL) under N_2 was added a solution of EDCI (2.45 g, 12.8 mmol) in anhydrous DCM (30 mL) dropwise over 45 minutes. The solution was stirred under N_2 at room temperature for 16 hours. The solution was diluted with DCM (250 mL), washed with aqueous NH_4Cl solution (2 × 150 mL) and water (100 mL), dried (Na_2SO_4), filtered and concentrated *in vacuo*. The crude mixture was purified by flash column chromatography (10% EtOAc in DCM) yielding eicosyl ester **14** (1.45 g, 36%) as a white solid: R_f 0.6 (20:80 EtOAc:hexane); HRMS (ESI) calc. for $[C_{54}H_{97}N_5O_6Na]^+$ 934.7331, found 934.7330; ^{1}H NMR (500 MHz, $CDCl_3$) δ 0.78 (3H, s, 18-H_3), 0.87 (3H, t, *J* 7.0, CH_2CH_3), 0.92 (3H, d, *J* 6.2, 21-H_3), 0.94 (3H, s, 19-H_3), 0.99 (1H, td, *J* 14.5, 3.5), 1.09 (1H, td, *J* 11.9, 5.6), 1.25 (34H, app. br s, $OCH_2CH_2(CH_2)_{17}CH_3$), 1.40 (9H, s, $C(CH_3)_3$), 1.41 (9H, s, $C(CH_3)_3$), 2.18 (1H, ddd, *J* 16.1, 10.9, 5.1), 2.40-2.58 (1H, m), 3.20-3.30 (1H, m, 3β-H), 3.63 (1H, br s, 7β-H), 3.95-4.03 (1H, m, 12β-H), 4.08 (2H, t, *J* 6.3, $CO_2CH_2CH_2(CH_2)_{17}CH_3$), 5.29 (1H, br s, 7α-NH), 5.50 (1H, br s, 12α-NH); ^{13}C NMR (126 MHz, $CDCl_3$) δ 13.8 (18-CH_3), 14.3 (CH_2CH_3), 17.6 (21-CH_3), 22.8 (CH_2), 23.1 (CH_2), 23.1 (19-CH_3), 26.4 (CH_2), 27.1 (CH_2), 27.8 (CH_2), 28.6 (CH_2), 28.7 ($C(CH_3)_3$), 28.7 ($C(CH_3)_3$), 28.8 (CH), 29.5 (CH_2), 29.5 (CH_2), 29.7 (CH_2), 29.7 (CH_2), 29.8 (CH_2), 29.8 (CH_2), 29.8 (CH_2), 30.7 (CH_2), 31.8 (CH_2), 32.0 (CH_2), 32.4 (CH_2), 34.9 (C), 35.4 (CH_2), 35.5 (CH_2), 35.7 (CH), 37.2 (CH), 41.9 (CH), 44.6 (CH), 44.8 (C), 47.4 (7-CH), 49.4 (CH), 53.3 (12-CH), 62.0 (3-CH), 65.5 ($OCH_2(CH_2)_{18}CH_3$), 78.8 ($C(CH_3)_3$), 78.9 ($C(CH_3)_3$), 155.7 (CONH), 155.7 (CONH), 175.9 ($CO_2CH_2(CH_2)_{18}CH_3$).

Eicosanoyl 3α-trifluoroacetamido-7α,12α-di[*N*-(*t*-butoxycarbonyl)amino]-5β-cholan-24-oate D

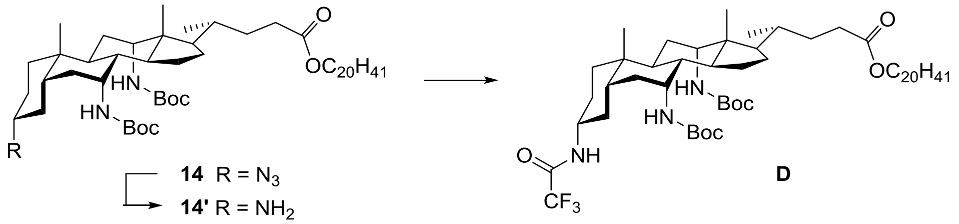

To a solution of 3α-azide **14** (1.42 g, 1.56 mmol) in acetic acid (100 mL) was added zinc dust (1.42 g, 100% by weight) and the mixture was stirred under N_2 for 48 hours. The zinc was removed by filtration and washed with acetic acid (3 × 30 mL) and the combined filtrates were concentrated *in vacuo*. The residue was redissolved in DCM (200 mL) and washed with saturated aqueous $NaHCO_3$ (2 × 50 mL). The organic phase was dried ($MgSO_4$), filtered and concentrated *in vacuo* yielding the crude 3α-amine **14'**: LRMS (ESI) calc. for $[C_{54}H_{100}N_3O_6]^+$ 886.76, found 886.77.

To a solution of 3α-amine **14'** in anhydrous DCM (90 mL) was added DIPEA (575 µL, 3.30 mmol) and TFAA (200 µL, 1.45 mmol) and the mixture was stirred under N_2 for 3 hours. The solvent was removed *in vacuo* and the residue was redissolved in DCM (150 mL) and washed with water (2 × 50 mL). The organic phase was dried ($MgSO_4$), filtered and concentrated *in vacuo*. The crude mixture was purified by flash column chromatography (7% EtOAc in DCM) yielding 3α-trifluoroamido eicosanoyl **D** (610 mg, 40% over 2 steps) as a white solid: R_f 0.5 (10% EtOAc in DCM); HRMS (ESI) calc. for $[C_{56}H_{98}F_3N_3O_7Na]^+$ 1004.7249, found 1004.7251; ^{1}H NMR (500 MHz, $CDCl_3$) δ 0.79 (3H, s, 18-H_3), 0.88 (3H, t, *J* 6.9, CH_2CH_3), 0.89 (3H, d, *J* 6.3, 21-H_3), 0.96 (3H, s, 19-H_3), 1.25 (34H, app. br s, $OCH_2CH_2(CH_2)_{17}CH_3$), 1.40 (9H, s, $C(CH_3)_3$), 1.41 (9H, s, $C(CH_3)_3$), 2.21 (1H, ddd, *J* 15.8, 9.5, 6.7), 2.34 (1H, ddd, *J* 15.3, 10.1, 4.9), 3.66 (2H, br s, 3β-H and 7β-H), 3.91 (1H br s, 12β-H), 4.04 (2H, t, *J* 6.8, $CO_2CH_2CH_2(CH_2)_{17}CH_3$), 4.61 (1H br s, 7α-NH), 4.82 (1H, br s, 12α-NH), 6.34 (1H, br s, 3α-NH); ^{13}C NMR (126 MHz, $CDCl_3$) δ 13.9 (18-CH_3), 14.3 (CH_2CH_3), 17.3 (21-CH_3), 22.8 (CH_2), 23.2 (CH_2), 23.2 (19-CH_3), 26.1 (CH_2), 26.7 (CH_2), 27.2 (CH_2), 27.3 (CH_2), 28.6 ($C(CH_3)_3$), 28.6 ($C(CH_3)_3$), 28.8 (CH_2), 29.4 (CH_2), 29.5 (CH_2), 29.6 (CH_2), 29.7 (CH_2), 29.8 (CH_2), 29.8 (CH_2), 28.8 (CH_2), 30.9 (CH_2), 31.6 (CH_2), 32.1 (CH_2), 34.7 (C), 35.0 (CH), 35.4 (CH_2), 35.7 (CH_2), 37.1 (CH), 41.8 (CH), 44.8 (C), 45.0 (CH), 47.4 (CH), 48.7 (CH), 50.6 (CH), 53.5 (12-CH), 64.7 ($OCH_2(CH_2)_{18}CH_3$), 79.8 ($C(CH_3)_3$), 116.0 (q, $J_{C\text{-}F}$ 288.5, $COCF_3$), 155.3 (CONH), 156.5 (q, $^2J_{C\text{-}F}$ 36.9, $COCF_3$), 174.4 ($CO_2CH_2(CH_2)_{18}CH_3$); ^{19}F NMR (470 MHz, $CDCl_3$) δ -76.0.

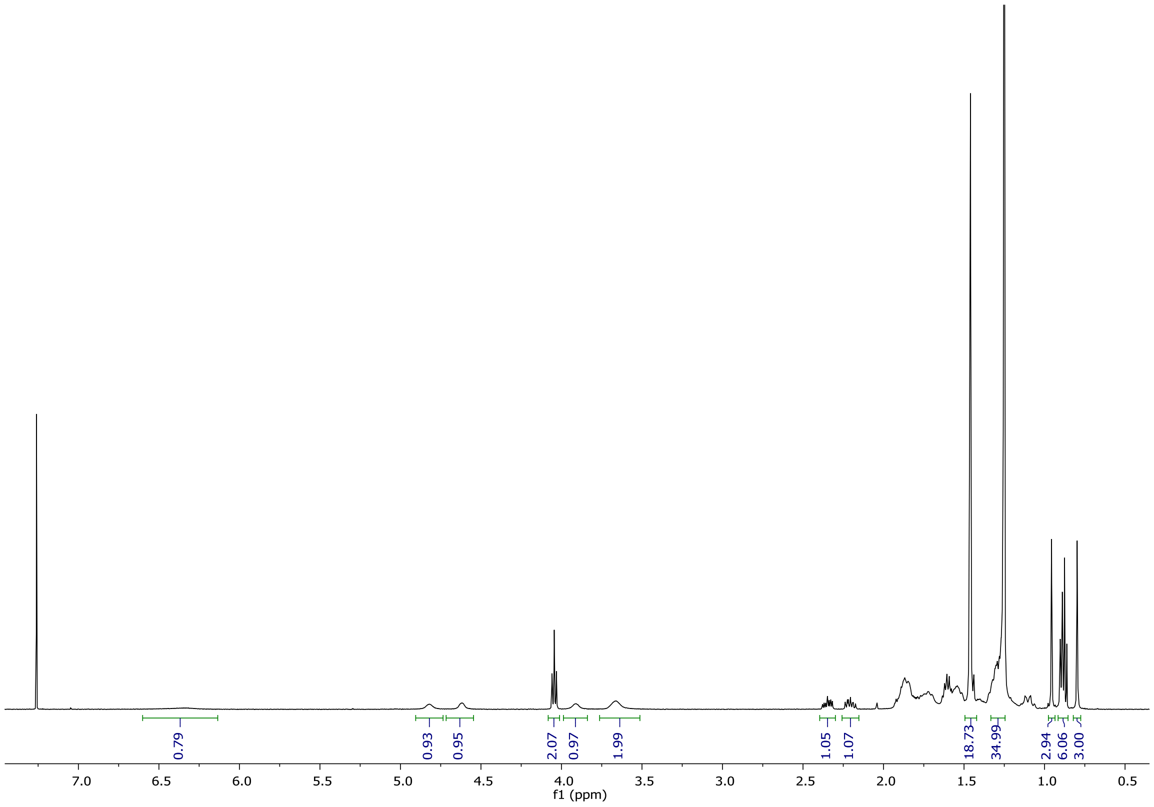

Figure S5. ^{1}H NMR spectrum of **D** in $CDCl_3$.

Cholapod receptor 10

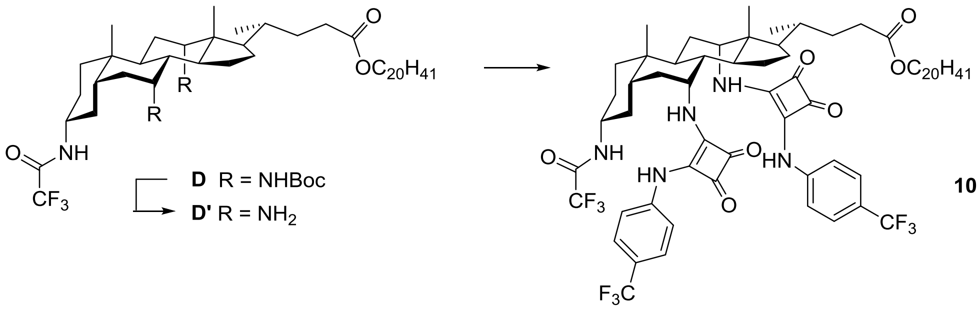

To a solution of Boc-protected diamine **D** (130 mg, 0.133 mmol) in anhydrous DCM (2.2 mL) was added TFA (1.8 mL) dropwise over 5 minutes. The solution was stirred at room temperature, under N_2, for 16 hours. The solvent was removed *in vacuo* and the residue redissolved in DCM (50 mL) and washed with saturated aqueous $NaHCO_3$ (2 × 30 mL). The aqueous phases were extracted with DCM (2 × 30 mL) and the combined organic extracts were dried (Na_2SO_4), filtered and concentrated *in vacuo* yielding the crude diamine **D'** (87 mg, 84%).

To a solution of diamine **D'** (87 mg, 0.11 mmol) in anhydrous MeOH (1 mL) was added squaramate **17** (90 mg, 0.33 mmol) and DIPEA (60 µL, 0.34 mmol). The resulting suspension was stirred at room temperature, under N_2, for 24 hours. The solvent was removed *in vacuo* and the crude residue was purified by flash column chromatography (5% MeOH in DCM). The obtained yellow material was dissolved in EtOAc (30 mL), washed with aqueous H_2SO_4 (15 mL, 0.5 M)[5] and water (15 mL), dried ($MgSO_4$), filtered and concentrated *in vacuo* yielding squaramide receptor **10** (68 mg, 48%) as a yellow solid: R_f 0.2 (10% MeOH in DCM); ν_{max} (neat)/cm^{-1} 3271 (NH), 2924 (CH), 2852 (CH), 1793 (C=O), 1704 (C=O), 1614, 1577, 1531, 1441, 1416, 1318, 1163, 1116, 1068, 1015, 836; HRMS (ESI) calc. for $[C_{68}H_{90}F_9N_5O_7Na]^+$ 1282.6595, found 1282.6589; elemental analysis $[C_{68}H_{90}F_9N_5O_7$ + $1.H_2O]$ calculated C 63.88, H 7.25, N 5.48, found C 64.23, H 7.29, N 5.42; NMR spectra were obtained on a solution of **10** (2 mM) in $CDCl_3$ to which $Et_4N^+Cl^-$ (2 eq) had been added: ^{1}H NMR (500 MHz, $CDCl_3$) δ 0.86 (3H, s, 18-H_3), 0.88 (3H, t, *J* 6.9, CH_2CH_3), 0.92 (3H, d, *J* 6.6, 21-H_3), 0.97 (3H, s, 19-H_3), 1.19-1.30 (34H, br m, $OCH_2CH_2(CH_2)_{17}CH_3$), 1.37 (12H, tt, *J* 7.3 and 1.7, Et_4N^+ CH_3), 2.30 (1H, ddd, *J* 15.1, 9.6 and 5.2), 2.35-2.52 (2H, m), 3.40 (q, *J* 7.3, Et_4N^+ CH_2), 3.76-3.88 (1H, m, 3β-H), 4.00 (2H, t, *J* 6.8, $CO_2CH_2CH_2(CH_2)_{17}CH_3$), 4.30 (1H, br s, 7β-H), 4.79-4.85 (1H, m, 12β-H), 7.19 (1H, d, *J* 8.8, 3α-N*H*), 7.58 (4H, app. t, *J* 7.8, 4 × ArH), 7.79-7.85 (5H, m, 4 × ArH and 12α-NH), 8.39 (1H, d, *J* 8.7, 7α-NH), 10.22 (1H, s, C-NH), 10.32 (1H, s, C-NH); ^{13}C NMR (126 MHz, $CDCl_3$) δ 7.8 (Et_4N^+ CH_3), 13.5 (18-CH_3), 14.3 (CH_2CH_3), 18.5 (21-CH_3), 22.1 (19-CH_3), 22.8 (CH_2), 23.2 (CH_2), 26.0 (CH_2), 26.5 (CH_2), 26.8 (CH_2), 28.5 (CH), 28.6 (CH_2), 28.8 (CH_2), 29.4

(CH_2), 29.5 (CH_2), 29.6 (CH_2), 29.7 (CH_2), 29.8 (CH_2), 30.7 (CH_2), 31.6 (CH_2), 32.1, 34.0, 34.3, 34.7, 35.4, 37.1 (CH), 41.2 (CH), 43.9 (CH), 45.6 (C), 48.9 (CH) 48.9 (3-CH), 52.4 (7-CH), 52.9 (Et_4N^+ CH_2), 58.1 (12-CH), 64.6 (O*C*$H_2(CH_2)_{18}CH_3$), 118.1 (ArCH), 118.2 (ArCH), 124.3 (Ar*C*CF_3), 124.6 (q, $J_{C\text{-}F}$ 271.4, ArC*C*F_3), 126.6 (ArCH), 142.7 (ArCNH), 156.4 (q, $^2J_{C\text{-}F}$ 36.9, *C*OCF_3), 164.8 (*C*=C), 164.9 (*C*=C), 168.8 (C=*C*), 169.9 (C=*C*), 174.3 (*C*$O_2CH_2(CH_2)_{18}CH_3$), 180.3 (CO), 181.1 (CO), 184.2 (CO), 184.4 (CO);[6] ^{19}F NMR (470 MHz, $CDCl_3$) δ -61.77, -61.79, -76.03.

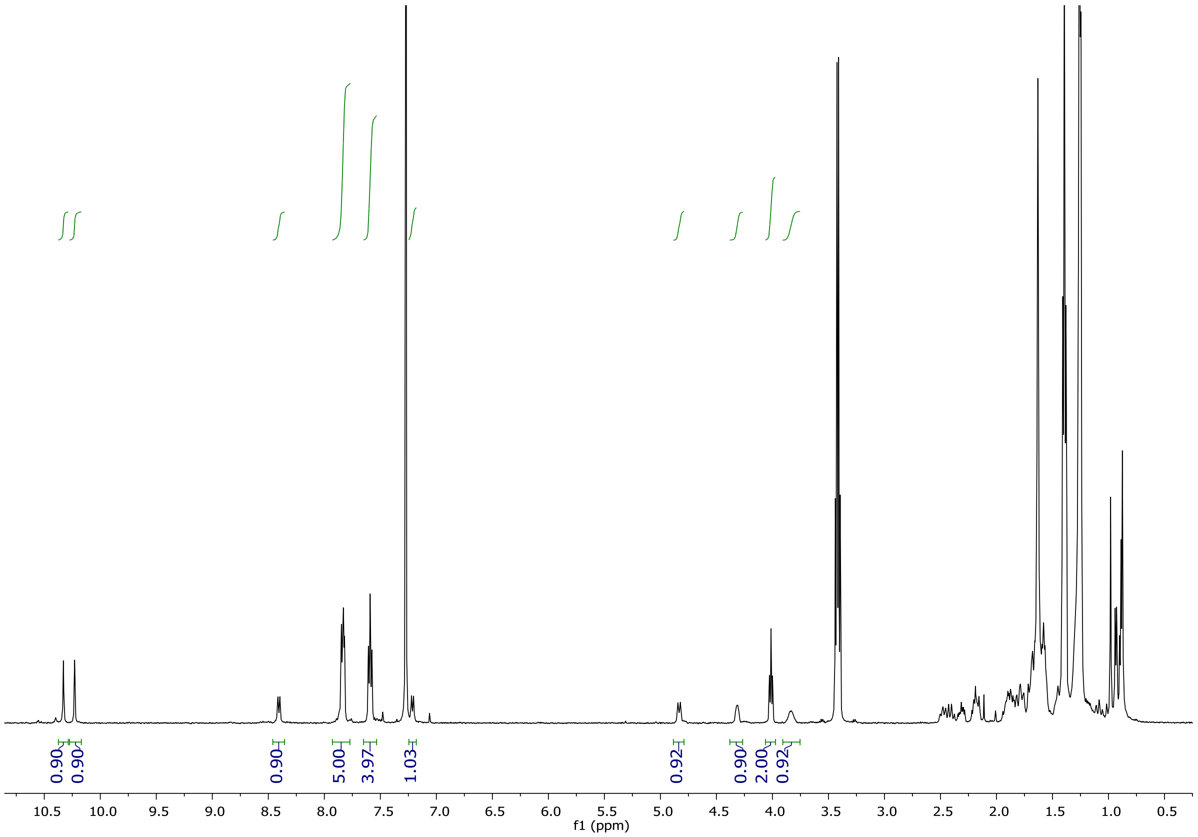

Figure S6. ^{1}H NMR spectrum of receptor **10** in $CDCl_3$ in the presence of 2 equivalents of $Et_4N^+Cl^-$.

Cholapod receptor 11

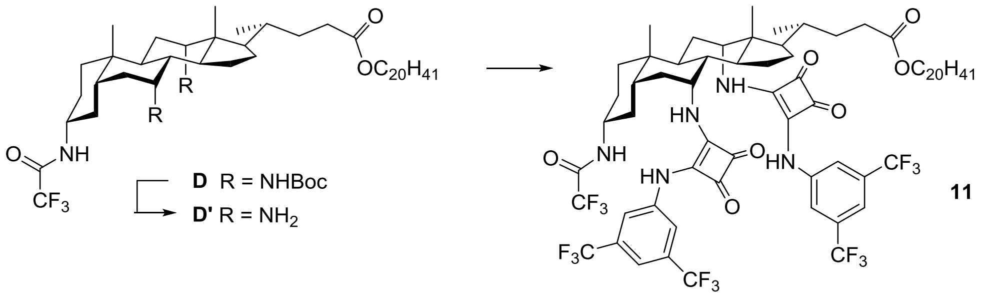

To a solution of Boc-protected diamine **D** (130 mg, 0.133 mmol) in anhydrous DCM (2.2 mL) was added TFA (1.8 mL) dropwise over 5 minutes. The solution was stirred at room temperature, under $N_{2,}$ for 16 hours. The solvent was removed *in vacuo* and the residue redissolved in DCM (50 mL) and washed with saturated aqueous $NaHCO_3$ (2 × 30 mL). The aqueous phases were extracted with DCM (2 × 30 mL) and the combined organic extracts were dried (Na_2SO_4), filtered and concentrated *in vacuo* yielding the crude diamine **D’** (89 mg, 86%).

To a solution of diamine **D’** (89 mg, 0.11 mmol) in anhydrous MeOH (1 mL) was added squaramate **18** (116 mg, 0.342 mmol) and DIPEA (60 µL, 0.34 mmol). The resulting suspension was stirred at room temperature, under N_2, for 24 hours. The solvent was removed *in vacuo* and the crude residue was purified by flash column chromatography (5% MeOH in DCM). The obtained yellow material was dissolved in EtOAc (30 mL), washed with aqueous H_2SO_4 (15 mL, 0.5 M)[5] and water (15 mL), dried ($MgSO_4$), filtered and concentrated *in vacuo* yielding squaramide receptor **11** (130 mg, 82%) as a yellow solid: R_f 0.2 (10% MeOH in DCM); ν_{max} (neat)/cm^{-1} 3263 (NH), 2923 (CH), 2852 (CH), 1792 (C=O), 1730 (C=O), 1677, 1603, 1574, 1527, 1502, 1439, 1364, 1241, 1173, 1081, 1027, 897, 751, 689, 661; HRMS (ESI) calc. for $[C_{70}H_{88}F_{15}N_5O_7Na]^+$ 1418.6292, found 1418.6336; elemental analysis [$C_{70}H_{88}F_{15}N_5O_7$ + 1.H_2O] calculated C 59.44, H 6.41, N 4.95, found C 59.23, H 6.44, N 4.86; NMR spectra were obtained on a solution of **11** (2 mM) in $CDCl_3$ to which $Et_4N^+Cl^-$ (2 eq) had been added: ^1H NMR (500 MHz, $CDCl_3$) δ 0.87 (3H, s, 18-H_3), 0.88 (3H, t, *J* 6.9, CH_2CH_3), 0.93 (3H, d, *J* 6.6, 21-H_3), 0.97 (3H, s, 19-H_3), 1.20-1.30 (34H, br m, $OCH_2CH_2(CH_2)_{17}CH_3$), 1.39 (12H, tt, *J* 7.3 and 1.7, Et_4N^+ CH_3), 2.30 (1H, ddd, *J* 15.1, 10.1 and 5.6), 2.33-2.46 (2H, m), 3.43 (q, *J* 7.3, Et_4N^+ CH_2), 3.78-3.89 (1H, m, 3β-H), 4.00 (2H, t, *J* 6.8, $CO_2CH_2CH_2(CH_2)_{17}CH_3$), 4.32 (1H, br s, 7β-H), 4.82-4.88 (1H, m, 12β-H), 7.15 (1H, d, *J* 8.9, 3α-N*H*), 7.47 (1H, s, ArH), 7.49 (1H, s, ArH), 7.74 (1H, d, *J* 11.0, 12α-NH), 8.21 (2H, s, 2 × ArH), 8.22 (2H, s, 2 × ArH), 8.35 (1H, d, *J* 8.9, 7α-NH), 10.18 (1H, s, C-NH), 10.39 (1H, s, C-NH); ^{13}C NMR (126 MHz, $CDCl_3$) δ 8.0 (Et_4N^+ CH_3), 13.5 (18-CH_3), 14.2 (CH_2CH_3), 18.3 (21-CH_3), 22.1 (19-CH_3), 22.8 (CH_2), 23.1 (CH_2), 26.0 (CH_2),

26.5 (CH_2), 27.1 (CH_2), 28.4 (CH), 28.5 (CH_2), 28.8 (CH_2), 29.4 (CH_2), 29.5 (CH_2), 29.6 (CH_2), 29.7 (CH_2), 29.8 (CH_2), 29.8 (CH_2), 30.7 (CH_2), 31.5 (CH_2), 32.0, 33.9, 34.1, 34.9, 35.3, 37.0 (CH), 41.0 (CH), 44.2 (CH), 45.4 (C), 48.9 (CH) 48.9 (3-CH), 52.4 (7-CH), 52.9 (Et_4N^+ CH_2), 58.1 (12-CH), 64.6 (O*C*$H_2(CH_2)_{18}CH_3$), 115.7 (ArCH), 118.3 (ArCH), 123.3 (q, $J_{C\text{-}F}$ 274.7, ArC*C*F_3), 132.9 (Ar*C*CF_3), 141.1 (ArCNH), 164.2 (*C*=C), 164.4 (*C*=C), 169.4 (C=*C*), 169.9 (C=*C*), 174.3 (*C*$O_2CH_2(CH_2)_{18}CH_3$), 180.3 (CO), 181.0 (CO), 184.2 (CO), 184.4 (CO);[6] ^{19}F NMR (470 MHz, $CDCl_3$) δ -63.35, -63.42, -76.59.

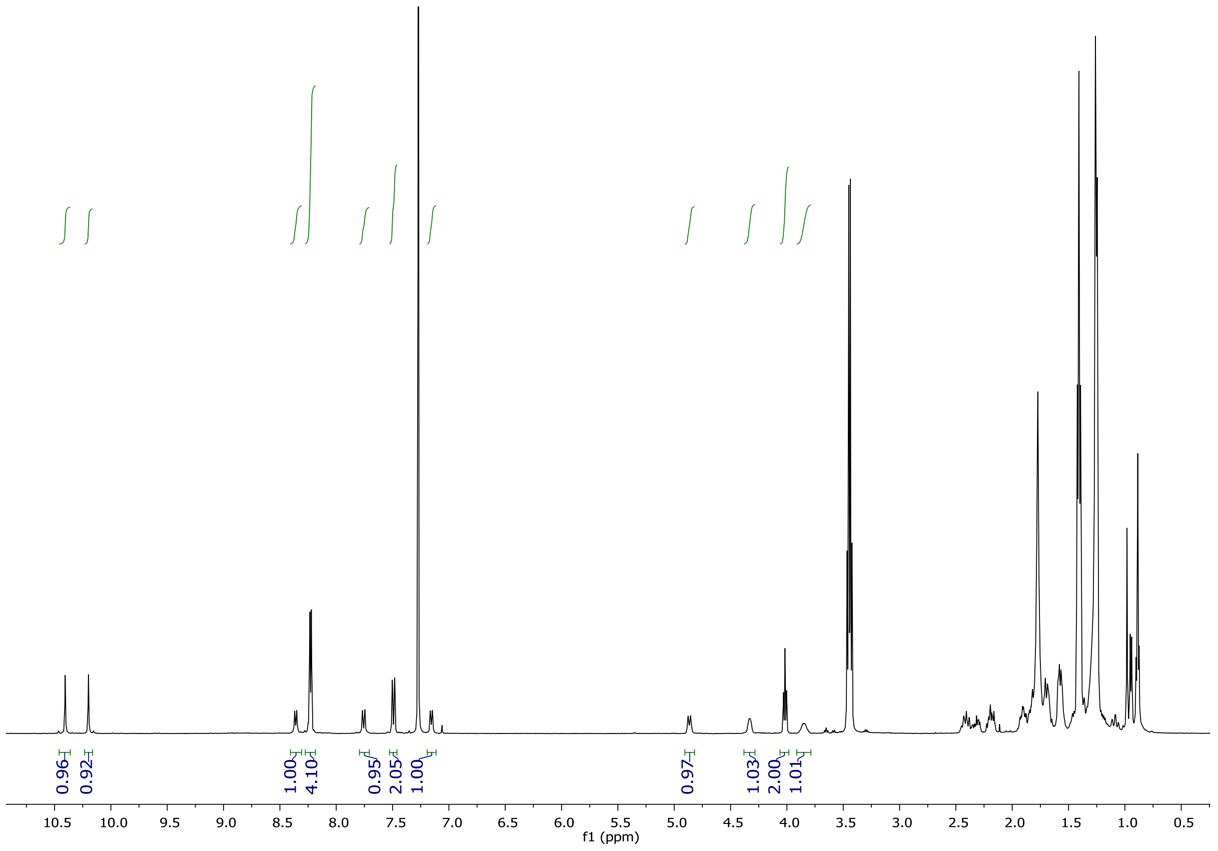

Figure S7. ^{1}H NMR spectrum of receptor **11** in $CDCl_3$ in the presence of 2 equivalents of $Et_4N^+Cl^-$.

2. Binding studies

Preparation of $Et_4N^+EtSO_3^-$ by neutralisation

While monitoring the pH with an electronic pH meter, an aqueous solution of $EtSO_3H$ was added dropwise to an aqueous solution of $Et_4N^+OH^-$ until pH 7.00 was observed. The resulting aqueous solution was concentrated i*n vacuo*, maintaining the temperature below 50 °C (this precaution was taken in order to prevent elimination of a Et_4N^+ ethyl group). The solution was further dried by lyophilisation yielding $Et_4N^+EtSO_3^-$ as a white solid.

General procedure for extraction studies in chloroform by Cram's extraction method [7,8]

$Et_4N^+EtSO_3^-$ was obtained as above, other Et_4N^+ salts were obtained from commercial suppliers. Due to their hygroscopic nature, the salts were dried overnight under high vacuum before use. Receptors were also dried under high vacuum overnight before use. All host solutions were prepared using chloroform that had been deacidified by passage through a flash chromatography column containing activated basic alumina. Guest solutions were prepared using deionised water that had been passed through a Millipore filtration system.

The following is a typical example of an extraction experiment involving receptor **11** and $Et_4N^+Cl^-$.

A solution of squaramide **11** (8.1×10^{-6} M) in deacidified chloroform was prepared and a known volume (100 mL) was added to a 1 L round bottomed flask. To this organic solution was added an aqueous solution of $Et_4N^+Cl^-$ (2.6×10^{-5} M, 500 mL). A magnetic stirring bar was added to the flask and the flask was immersed in a water bath that was heated to 303 K. After 30 s the flask was stoppered and the contents were stirred vigorously. After 30 minutes stirring was stopped and the two phases were allowed to separate. The majority of the aqueous phase was removed by decantation and the remaining mixture was poured into a separating funnel. The organic phase was separated and filtered through Whatman 1PS hydrophobic filter paper to remove any trace of aqueous phase. The filtrate was concentrated *in vacuo* and the resulting solid was dried on a high vacuum line. The solid was then dissolved in acetone-d_6 and an excess of tetraphenylphosphonium bromide (TPhP-Br, ~7.5 mM) was added to sharpen peaks and facilitate integration. A ^1H NMR spectrum was collected at 298 K and referenced to the residual solvent peak (δ = 2.05 ppm). The

CH_2 signal of $Et_4N^+Cl^-$ was integrated with respect to the receptor signals to give the guest:host ratio (*R*), allowing the value of K_a to be determined (see equations below).

For the strongest receptors (**8-11**) the concentration of host solution used was between 7 × 10^{-6} and 8 × 10^{-6} M. The volume of host solution used was 100 mL. The concentration of guest solution was varied in order to give R values in the range 0.1 - 0.7, avoiding saturation and allowing accurate integration. The volume of guest solution used was 500 mL, ensuring that only a small fraction of guest was extracted in to the organic phase.

For the weaker receptors (**6** and **7**) less dilute conditions were required. The concentration of host solution was kept at approximately 3 × 10^{-4} M and the volume of host solution used was 3 mL. The volume of guest solution used was 10 mL and the concentration was again varied to give appropriate *R* values.

The value for *R* obtained from the ^{1}H NMR spectra was used to calculate the extraction equilibrium constant K_e:

$$H_{org} + X^-_{aq} + Y^+_{aq} \overset{K_e}{\rightleftharpoons} HX^-Y^+_{org} \qquad K_e = \frac{[HXY]_{org}}{[H]_{org}[X^-]_{aq}[Y^+]_{aq}}$$

$$K_e = \frac{R}{(1-R)\left([G]_{aq}^{initial} - R\frac{V_{org}}{V_{aq}}[H]_{org}^{initial}\right)^2}$$

We have to take into account the equilibrium between the unbound $Et_4N^+X^-$ guest in the organic phase and the dissociated Et_4N^+ and X^- ions in the aqueous phase, described by:

$$X^-_{aq} + Y^+_{aq} \overset{K_d}{\rightleftharpoons} X^-Y^+_{org} \qquad K_d = \frac{[X^-Y^+]_{org}}{[X^-]_{aq}[Y^+]_{aq}}$$

Values for K_d have been determined and reported previously to be 1.269 × 10^{-5} M^{-1} for $Et_4N^+Cl^-$, 2.183 × 10^{-4} M^{-1} for $Et_4N^+Br^-$, 8.367 × 10^{-3} M^{-1} for $Et_4N^+I^-$, 1.913 × 10^{-4} M^{-1} for $Et_4N^+NO_3^-$, 8.540 × 10^{-7} M^{-1} for $Et_4N^+OAc^-$, 1.080 × 10^{-2} M^{-1} for $Et_4N^+ClO_4^-$ and 2.849 × 10^{-5} M^{-1} for $Et_4N^+EtSO_3^-$.[8]

The binding constant K_a is then calculated from K_e and K_d :

$$H_{org} + X^-Y^+_{org} \overset{K_a}{\rightleftharpoons} HX^-Y^+_{org} \qquad K_a = \frac{[HXY]_{org}}{[H]_{org}[X^-Y^+]_{org}} = \frac{K_e}{K_d}$$

K_a values for chloride anion binding by extraction method

Table S1. Extraction data and derived association constants of squaramides receptors **6-11** to $Et_4N^+Cl^-$ in $CHCl_3$.

Receptor	[H] (M)	[G] (M)	V_{org} (mL)	V_{aq} (mL)	R	K_a (M^{-1})
**6**	2.8×10^{-4}	1.5×10^{-4}	3.0	10.0	0.584	5.27×10^{10}
	2.8×10^{-4}	2.0×10^{-4}	3.0	10.0	0.675	4.34×10^{10}
					**Average:**	**4.8×10^{10}**
**7**	3.1×10^{-4}	3.2×10^{-4}	3.0	10.0	0.168	1.69×10^{11}
	3.1×10^{-4}	4.0×10^{-4}	3.0	10.0	0.226	1.61×10^{11}
					**Average:**	**1.6×10^{11}**
**8**	7.5×10^{-6}	2.1×10^{-5}	100.0	500.0	0.186	2.97×10^{13}
	7.5×10^{-6}	2.6×10^{-5}	100.0	500.0	0.238	2.78×10^{13}
					**Average:**	**2.9×10^{13}**
**9**	7.8×10^{-6}	2.6×10^{-5}	100.0	500.0	0.287	4.84×10^{13}
	7.8×10^{-6}	3.1×10^{-5}	100.0	500.0	0.329	4.09×10^{13}
					**Average:**	**4.5×10^{13}**
**10**	7.6×10^{-6}	2.1×10^{-5}	100.0	500.0	0.186	4.25×10^{13}
	7.6×10^{-6}	2.6×10^{-5}	100.0	500.0	0.238	3.73×10^{13}
					**Average:**	**4.0×10^{13}**
**11**	8.1×10^{-6}	2.6×10^{-5}	100.0	500.0	0.521	1.35×10^{14}
	8.1×10^{-6}	3.1×10^{-5}	100.0	500.0	0.553	1.05×10^{14}
					**Average:**	**1.2×10^{14}**

K_a values for binding to various anions by extraction method

Table S2. Extraction data and derived association constants of squaramide receptor **11** to various anions (as the Et_4N^+ salt) in $CHCl_3$.

Anion	[H] (M)	[G] (M)	V_{org} (mL)	V_{aq} (mL)	R	K_a (M^{-1})
Cl^-	8.1×10^{-6}	2.6×10^{-5}	100.0	500.0	0.521	1.35×10^{14}
	8.1×10^{-6}	3.1×10^{-5}	100.0	500.0	0.553	1.05×10^{14}
					**Average:**	$\mathbf{1.2 \times 10^{14}}$
Br^-	7.5×10^{-6}	1.5×10^{-5}	100	500.0	0.435	1.68×10^{13}
	7.5×10^{-6}	2.0×10^{-5}	100	500.0	0.544	1.45×10^{13}
					**Average:**	$\mathbf{1.6 \times 10^{13}}$
I^-	7.7×10^{-6}	1.1×10^{-5}	100.0	500.0	0.284	4.42×10^{11}
	7.7×10^{-6}	2.2×10^{-5}	100.0	500.0	0.543	3.30×10^{11}
					**Average:**	$\mathbf{3.9 \times 10^{11}}$
NO_3^-	7.5×10^{-6}	1.5×10^{-5}	100.0	500.0	0.343	1.27×10^{13}
	7.5×10^{-6}	2.0×10^{-5}	100.0	500.0	0.555	1.73×10^{13}
					**Average:**	$\mathbf{1.5 \times 10^{13}}$
AcO^-	6.8×10^{-6}	7.0×10^{-5}	100.0	500.0	0.599	3.61×10^{14}
	6.8×10^{-6}	9.4×10^{-5}	100.0	500.0	0.709	3.31×10^{14}
					**Average:**	$\mathbf{3.5 \times 10^{14}}$
ClO_4^-	7.3×10^{-6}	4.1×10^{-5}	100.0	500.0	0.339	2.86×10^{10}
	7.3×10^{-6}	5.2×10^{-5}	100.0	500.0	0.370	2.09×10^{10}
					**Average:**	$\mathbf{2.5 \times 10^{10}}$
$EtSO_3^-$	7.1×10^{-6}	3.1×10^{-5}	100.0	500.0	0.306	1.77×10^{13}
	7.1×10^{-6}	4.7×10^{-5}	100.0	500.0	0.474	2.04×10^{13}
					**Average:**	1.91×10^{13}

^{1}H NMR titration studies

^{1}H NMR titrations of bis-squaramide receptors vs. $Bu_4N^+Cl^-$ were carried out in $CDCl_3$, in order to confirm a 1:1 binding stoichiometry. As expected, the plots indicated that binding was too strong for quantitative analysis (see Figures S9, S11).

$Bu_4N^+Cl^-$ was obtained from commercial suppliers. Host and guest solutions were prepared using $CDCl_3$ that had been deacidified by passage through a flash chromatography column containing basic alumina. The hygroscopic guest $Bu_4N^+Cl^-$ and the host compounds were dried under high vacuum prior to solution preparation. NMR titrations were performed by addition of aliquots of a $Bu_4N^+Cl^-$ solution to a solution of the receptor (**10**: 0.3 mM; **11**: 0.2 mM) in $CDCl_3$. The guest solutions were prepared by dissolving $Bu_4N^+Cl^-$ in a solution of host, so that the concentration of host did not decrease over the course of the experiment. Spectra were recorded on a Varian 500B spectrometer (proton sensitive probe) and referenced to the residual solvent peak in $CDCl_3$ (δ = 7.26 ppm).

^{1}H NMR titrations were also attempted in DMSO-d_6 + 0.5% H_2O with the aim of obtaining association constants in this medium. However the initial spectra were too broad to obtain accurate peak positions, and hence the data could not be fitted to a binding isotherm.

^{1}H NMR titration of receptor 10 with $Bu_4N^+Cl^-$ in $CDCl_3$

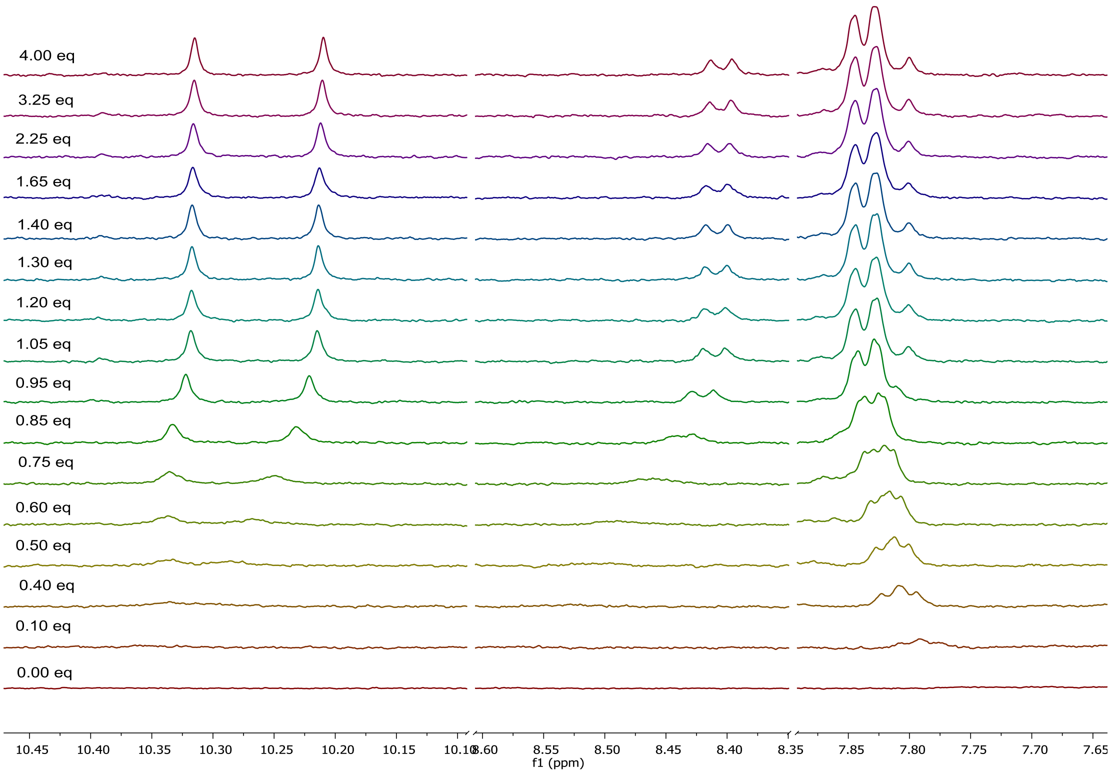

Figure S8. ^{1}H NMR spectra (ArH and ArNH signals) from the titration of $Bu_4N^+Cl^-$ into squaramide receptor **10** (0.3 mM) in $CDCl_3$ at 298 K. The amount of $Bu_4N^+Cl^-$ added is shown in equivalents relative to **10**.

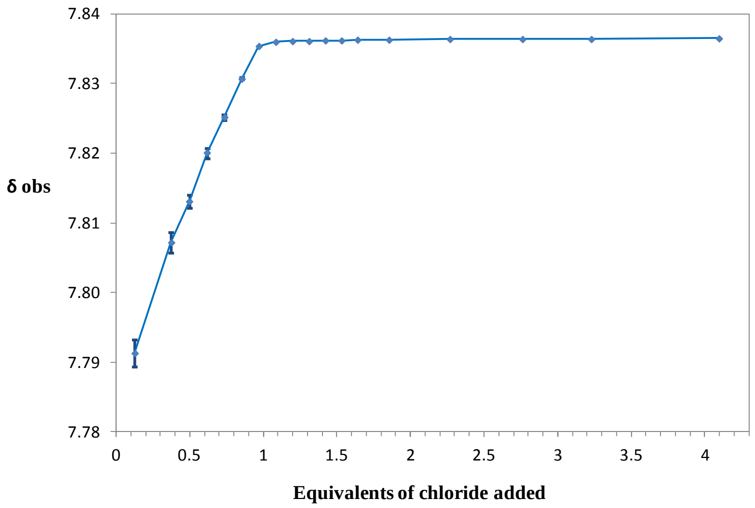

Figure S9. Graph showing the change in δ_{obs} for squaramide receptor **10** when titrated against $Bu_4N^+Cl^-$ in $CDCl_3$ at 298 K (ArH signal, δ = 7.78 ppm). Error bars represent the error in δ_{obs} due to peak broadening. No further change occurs after the addition of 1 equivalent of $Bu_4N^+Cl^-$ suggesting the formation of a 1:1 host:guest complex in $CDCl_3$.

^{1}H NMR titration of receptor 11 with $Bu_4N^+Cl^-$ in $CDCl_3$

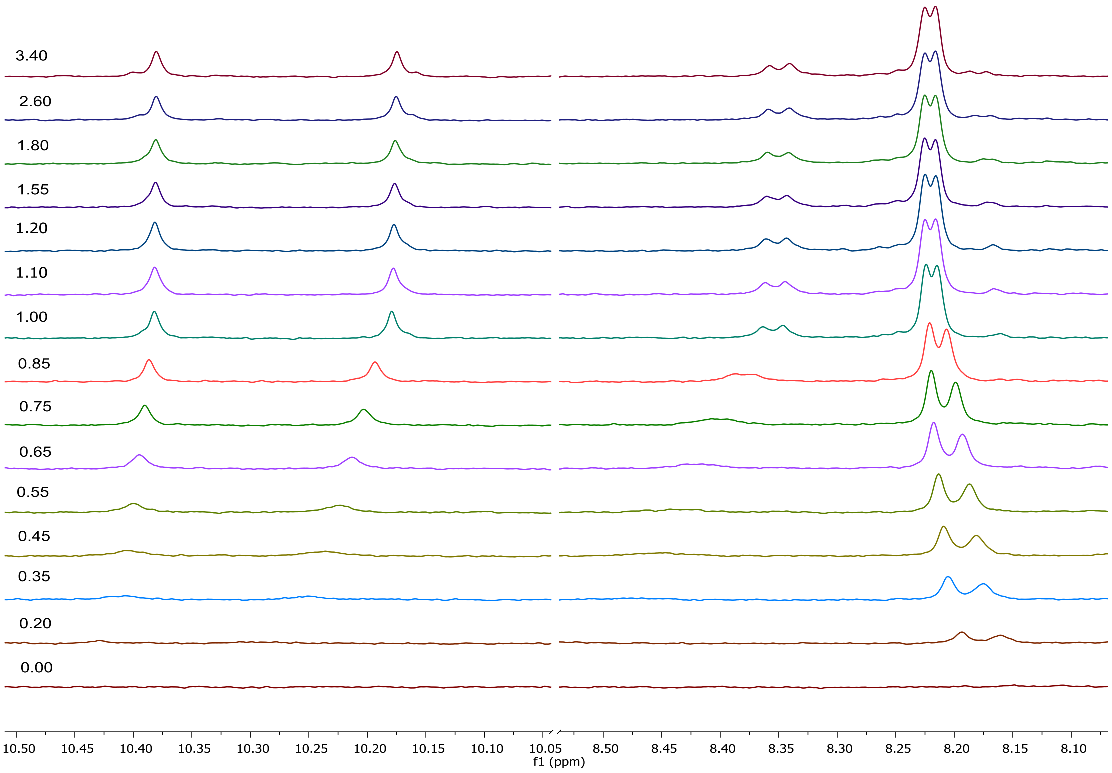

Figure S10. ^{1}H NMR spectra (ArH and ArNH signals) from the titration of $Bu_4N^+Cl^-$ into squaramide receptor **11** (0.2 mM) in $CDCl_3$ at 298 K. The amount of $Bu_4N^+Cl^-$ added is shown in equivalents relative to **11**.

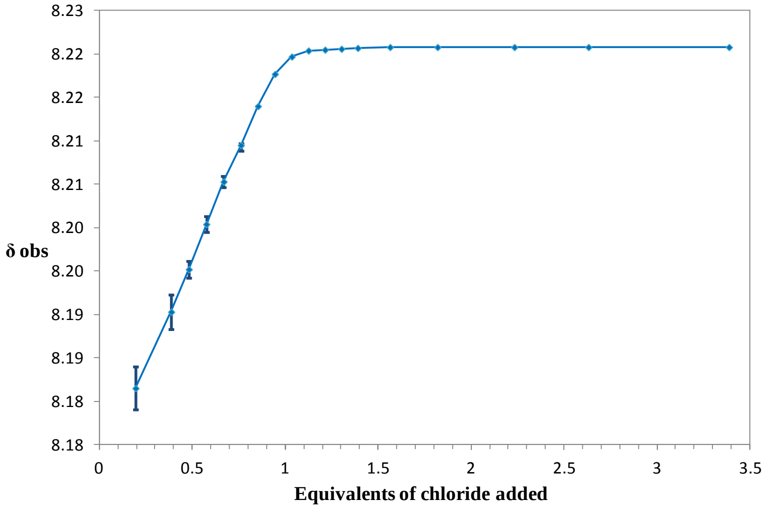

Figure S11. Graph showing the change in δ_{obs} for squaramide receptor **11** when titrated against $Bu_4N^+Cl^-$ in $CDCl_3$ at 298 K (ArH signal, δ = 8.17 ppm). Error bars represent the error in δ_{obs} due to peak broadening. No further change occurs after the addition of 1 equivalent of $Bu_4N^+Cl^-$ suggesting the formation of a 1:1 host:guest complex in $CDCl_3$.

3. Transport Studies

General procedure for transport measurements

Chloride ion transport was measured using large unilamellar vesicles (LUVs, 200 nm average diameter) composed of 1-palmitoyl-2-oleoyl-sn-glycero-3-phosphocholine (POPC) and cholesterol at a ratio of 7:3. POPC was obtained from Avanti® Polar Lipids, Inc. Extrusion apparatus and 200 nm polycarbonate membranes, were obtained from GC Technology Ltd. All lipid and receptor solutions were prepared using chloroform that had been purified by passage through basic alumina, and all aqueous solutions were prepared using deionised water that had been passed through a Millipore filtration system.

The following is a typical example of an anion transport experiment involving bis-squaramide receptor **8**:

The receptor (35.9 µL, 67.0 µM in deacidified $CHCl_3$ and HPLC MeOH; ~50:50 v/v) was added to solutions of POPC (358 µL, 11.7 mM in deacidified $CHCl_3$) and cholesterol (214 µl, 8.43 mM in deacidified $CHCl_3$) to give a receptor to lipid ratio of 1:2500. The solvents were evaporated under a gentle stream of N_2 and dried under high vacuum for 1 h. The resulting residue was hydrated with 500 µL of an aqueous solution of lucigenin (0.8 mM) and $NaNO_3$ (225 mM) and then sonicated for 30 s and stirred for 1 h to give heterogeneous LUVs. The multilamellar vesicles were disrupted by 10 freeze-thaw cycles and then the solution was carefully extruded (29 times) through a polycarbonate membrane (200 nm pore size) to give a uniform distribution of LUVs. The external lucigenin was removed by passing the solution through a size exclusion column (~2 g Sephadex 50G, eluting with $NaNO_3$ aq, 225 mM) and the collected vesicles were diluted to a volume of 15 mL (0.4 mM lipid) with $NaNO_3$ solution (225 mM). NaCl (75 µL, 1.0 M in 225 mM $NaNO_3$) was added with stirring to a quartz cuvette containing 3.00 mL of the vesicle solution and the fluorescence was monitored over 900 s at 25 °C using an excitation wavelength of 450 nm and an emission wavelength of 535 nm.

Half-lives of fluorescence decay were obtained by fitting the reciprocal transport curves (F_0/F) from 0-500 seconds to a single exponential decay function (Eq. 1) using Origin 9.0. The half-life was calculated using fit parameter 'b' (Eq. 2).

(1) $$\frac{F_0}{F} = y - ae^{-bt}$$

(2) $$t_{1/2} = \frac{\ln(2)}{b}$$

Initial rates of fluorescence decay were obtained by fitting the reciprocal transport curves from 0-500 seconds to a double exponential decay function (Eq. 3) using Origin 9.0, and differentiating at t = 0 (Eq. 4).

(3) $$\frac{F_0}{F} = y - ae^{-bt} - ce^{-dt}$$

(4) $$Initial\,rate = ab + cd$$

Chloride transport into vesicles by eicosyl ester squaramides 6-11

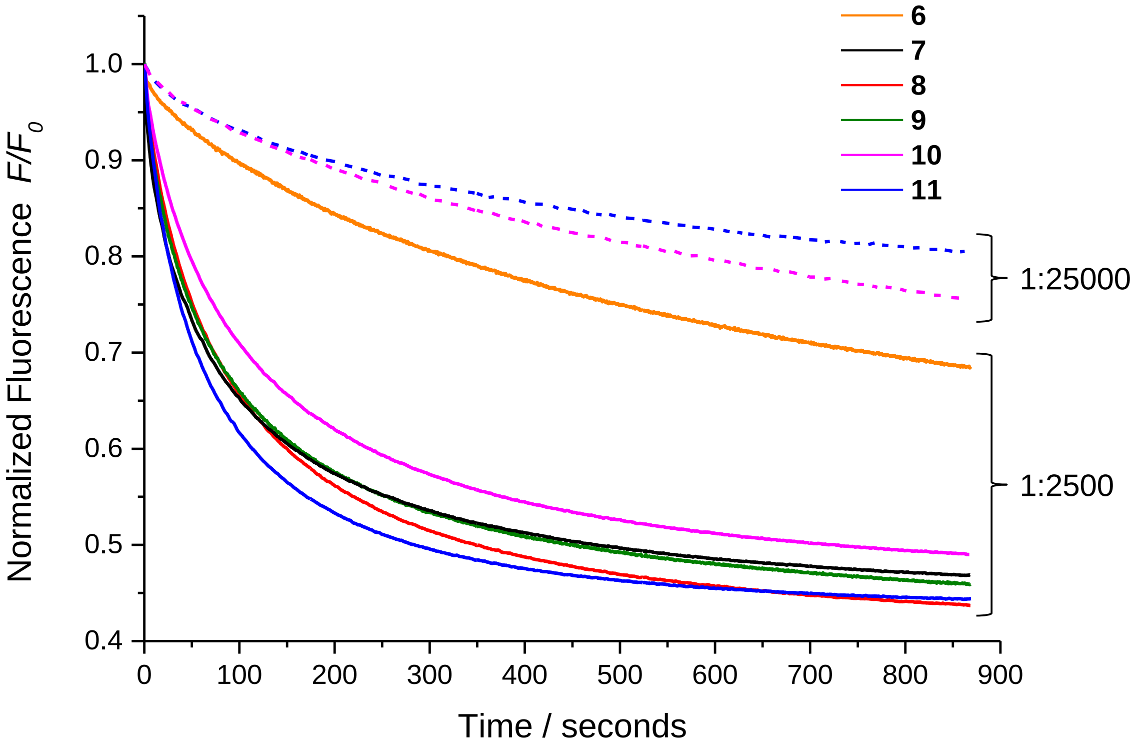

Figure S12. Chloride transport into 200 nm vesicles by squaramide receptors **6-11** at a receptor:lipid ratio of 1:2500, and by squaramides **10** and **11** at 1:25000 (broken lines). Transport rates at 1:25000 are roughly 10 times lower than those at 1:2500, suggesting that the relatively poor transport by **10** and **11** is not caused by self-association.

Table S3. Half-lives and initial rates of chloride transport into 200 nm vesicles by receptors **6-11**, at receptor:lipid ratios of 1:2500.

Receptor	Half-life (s)	Initial rate (s^{-1})
**6**	361	0.0020
**7**	122	0.0096
**8**	141	0.0082
**9**	128	0.0090
**10**	149	0.0065
**11**	106	0.0112

References and Notes

[1] V. del Amo, L. Siracusa, T. Markidis, B. Baragaña, K. M. Bhattarai, M. Galobardes, G. Naredo, M. N. Pérez-Payán, A. P. Davis, *Org. Biomol. Chem.* **2004**, *2*, 3320-3328.

[2] T. N. Lambert, J. M. Boon, B. D. Smith, M. N. Pérez-Payán, A. P. Davis, *J. Am. Chem. Soc.* **2002**, *124*, 5276-5277.

[3] A. J. Ayling, M. N. Pérez-Payán, A. P. Davis, *J. Am. Chem. Soc.* **2001**, *123*, 12716-12717.

[4] W. Yang, D. M. Du, *Org. Lett.* **2010**, *12*, 5450-5453; S. P. Kumar, P. M. C. Glória, L. M. Gonçalve, J. Gut, P. J. Rosenthal, R. Moreira, M. M. M. Santos, *Med. Chem. Commun.* **2012**, *3*, 489-493.

[5] Washing with aqueous H_2SO_4 removes DIPEA which is retained by the bis-squaramides during chromatography.

[6] The ^{13}C NMR peak due to CF$_3$ could not be observed, presumably due to splitting by 3 × F.

[7] E. P. Kyba, R. C. Helgeson, K. Madan, G. W. Gokel, T. L. Tarnowski, S. S. Moore, D. J. Cram, *J. Am. Chem. Soc.* **1977**, *99*, 2564-2571; A. J. Ayling, S. Broderick, J. P. Clare, A. P. Davis, M. N. Pérez-Payán, M. Lahtinen, M. J. Nissinen, K. Rissanen, *Chem. Eur. J.* **2002**, *8*, 2197.

[8] J. P. Clare, A. J. Ayling, J. B. Joos, A. L. Sisson, G. Magro, M. N. Pérez-Payán, T. N. Lambert, R. Shukla, B. D. Smith, A. P. Davis, *J. Am. Chem. Soc.* **2005**, *127*, 10739-10746.
